# Glial-Dopamine crosstalk: Astrocytic and microglial gatekeepers of neuroinflammation, plasticity, and motivation

**DOI:** 10.3934/Neuroscience.2026004

**Published:** 2026-02-04

**Authors:** Moawiah M Naffaa

**Affiliations:** Independent Researcher, Mountain View, CA 94040, USA

**Keywords:** glial-da crosstalk, astrocyte-microglia interactions, neuroinflammation, dopaminergic circuits, motivation and stress, PD, precision neurotherapeutics, single-cell and spatial transcriptomics

## Abstract

Dpamine (DA) signaling has long been framed through a neuron-centric lens; yet, mounting evidence reveals that glial cells (astrocytes and microglia) serve as indispensable gatekeepers of dopaminergic tone, synaptic plasticity, and neuroimmune balance. Single-cell, spatial, and optical imaging studies have redefined DA circuits as multicellular ecosystems in which glial receptors, transporters, and gliotransmitters dynamically sculpt neuromodulation and behavior. Astrocytes fine-tune DA clearance, glutamate buffering, and metabolic coupling, while microglia integrate immune and stress cues recalibrate dopaminergic signaling across striatal and cortical circuits. Their bidirectional interactions, both glia-glia and glia-neuron, mediate resilience or vulnerability in contexts ranging from motivation and stress adaptation to Parkinson's disease (PD), depression, and post-viral fatigue syndromes. In this review, we synthesized emerging evidence that glial-DA crosstalk is a systems-level regulator of neuroinflammation and plasticity, bridging cellular metabolism, immune tone, and behavioral output. By integrating multi-omics, in vivo imaging, and computational models, we proposed a translational framework for targeting astrocytic and microglial states to restore dopaminergic homeostasis. Understanding and manipulating these non-neuronal interfaces may open the next frontier in precision neuropsychiatry and neurodegeneration therapeutics.

## 1. Introduction

For decades, dopamine (DA) has been understood primarily through a neuronal lens. Synthesized and released by midbrain dopaminergic neurons, classically in the substantia nigra pars compacta and ventral tegmental area, DA was framed as a transmitter that sculpts basal ganglia, prefrontal cortex (PFC), amygdala, and interconnected circuits [1],[2]. Within this model, DA mediated a wide spectrum of functions, from motor control and reinforcement learning to motivation, decision-making, and working memory [3]. Pathological perturbations in this system were accordingly linked to movement disorders such as Parkinson's disease (PD), as well as psychiatric syndromes, including schizophrenia, addiction, and (attention-deficit/hyperactivity disorder) ADHD [4]. Glia, in contrast, were relegated to supportive or defensive roles: Astrocytes as ionic and metabolic buffers and microglia as immune sentinels engaged in pruning and injury repair [5].

This classical dichotomy has increasingly eroded over the past decade, as mounting evidence has repositioned astrocytes and microglia from peripheral bystanders to active regulators of dopaminergic tone. Astrocytes not only express functional DA receptors but also directly modulate synaptic plasticity. For tripartite synapseinstance, activation of D1/D5 receptors in spinal astrocytes has been shown to induce non-Hebbian long-term potentiation at primary afferent inputs, thereby reshaping excitatory drive independently of canonical neuronal pathways [6]. Complementing this, researchers identified astrocytic Oxytocin Receptor (OTR)-Dopamine D2 Receptor (D2) signaling as a potent regulator of neighboring dopaminergic neuron excitability, situating astrocytes within closed feedback loops that dynamically govern DA release [7]. Collectively, these findings converge on the recognition that astrocytic responses to DA are highly context-dependent, exerting anti-inflammatory and neuroprotective effects in some settings while promoting pro-inflammatory and neurotoxic outcomes in others, with gliotransmitter and neurotrophin release serving as key modulatory levers [8],[9].

Astrocytic influence also extends indirectly through crosstalk with other neuromodulators. Striatal astrocytes regulate extracellular GABA and adenosine, thereby constraining DA release through GABAA/B and adenosine A1 receptor pathways [10]. Notably, soma-to-soma configurations with cholinergic interneurons enable astrocytes to exert subsecond precision over DA dynamics, positioning them as fast integrators rather than slow homeostatic buffers.

Microglia have likewise emerged as indispensable architects of dopaminergic circuitry. Developmental studies demonstrate that microglia orchestrate DA axon growth, pruning, and synaptic connectivity, with early-life stress reprogramming microglial transcriptional states and destabilizing the maturation of dopaminergic projections [11],[12]. In adulthood, DA itself exerts reciprocal control over microglial activity. Experimental evidence shows that DA exposure activates inflammasome signaling and upregulates (interleukin-1β) IL-1β expression in microglia and macrophages, with the magnitude and direction of these responses determined by the relative balance of D1-like versus D2-like receptor expression [13]. Under conditions of inflammatory comorbidity, such as HIV infection, these effects are markedly amplified, establishing bidirectional feedback loops in which DA modulates microglial state, while activated microglia, in turn, regulate DA synthesis, reuptake, and neuronal survival.

Together, these findings force a conceptual shift. The once dominant tripartite synapse (neuron, astrocyte, and presynaptic terminal) must be expanded into a quadripartite model of dopaminergic regulation that integrates microglial, vascular, and immune influences [14]. In this expanded framework, dopaminergic signaling is governed by four interacting elements: Dopaminergic neurons, astrocytes, microglia, and the associated vascular and immune microenvironment, which together form a tetrasynaptic regulatory unit integrating neuromodulatory, metabolic, and inflammatory signals. Cytokine release, blood brain barrier dynamics, and immune trafficking emerge as indispensable modulators of dopamine physiology. High resolution single cell and spatial transcriptomic analyses reveal marked heterogeneity across astrocytic and microglial populations in midbrain and striatal territories, with some subpopulations enriched for dopamine receptor expression and others correlated with selective neuronal vulnerability in aging and disease [15]. These findings dismantle the notion of dopamine as a purely neuronal currency, reframing it as a network level signal embedded within glial, metabolic, and immune landscapes [16].

This reconceptualization yields three transformative implications: First, glial responses to DA are bidirectional and context-sensitive; receptor subtype, developmental window, and stress or disease state determine whether outcomes are neuroprotective or neurotoxic [17]. Second, DA-glia interactions exhibit striking temporal and spatial heterogeneity, differing across striatum, PFC, and hippocampus, and shifting from development to pathology [18]. Third, DA signaling is now inseparable from immune and metabolic states, embedding neuromodulation within vascular and systemic physiology [19].

The implications of these converging findings are profound and demand systematic synthesis. Recasting astrocytes and microglia as central gatekeepers of dopaminergic tone provides a novel conceptual lens through which to understand the mechanisms of selective vulnerability across a spectrum of disorders, including PD, depression, and schizophrenia. At the translational interface, the modulation of glial DA receptors, the fine-tuning of receptor subtype ratios, and the targeting of dopamine-sensitive inflammatory cascades emerge as promising therapeutic strategies. In this context, we seek to advance an integrative framework encompassing molecular, cellular, and systems-level perspectives within an expanded quadripartite synapse model designed to orient future research trajectories and inform the rational development of next-generation dopaminergic interventions.

We argue that astrocytes and microglia regulate dopaminergic vulnerability in PD and related neurodegenerative conditions through defined mechanisms including receptor-specific dopamine sensing, gliotransmitter and cytokine release, metabolic coupling, and feed-forward inflammatory loops that reshape dopaminergic circuit function.

## 2. Astrocytic control of DA signaling

With this framework in place, we first examine how astrocytes shape dopaminergic signaling through receptor-mediated sensing, gliotransmission, and circuit-specific modulation.

DA signaling is mediated by five G protein-coupled receptors (D1–D5), classically grouped into D1-like receptors (D1, D5), which stimulate adenylate cyclase via Gs proteins, and D2-like receptors (D2, D3, D4), which inhibit cyclic AMP production through Gi/o signaling [20]. These receptor classes differ in affinity, intracellular signaling kinetics, and circuit-level functions, enabling dopamine to exert phasic and tonic control across neural and glial populations.

Astrocytes have increasingly been recognized as circuit-defining regulators of dopaminergic neurotransmission. Far from being passive support elements, they express functional DA receptors, release gliotransmitters that shape synaptic plasticity, and buffer neuromodulators such as adenosine and GABA with subsecond precision, particularly within striatal networks [6],[10],[21],[22]. This dynamic control reframes dopaminergic function, positioning astrocytes as determinants of phasic versus tonic DA signaling and linking regional astrocytic heterogeneity to behavioral domains, including movement, reward, affect, and nociception (Table 1) [9].

**Table 1.** Astrocytic modulation of dopaminergic signaling.

Focus	Astrocytic Mechanisms	Circuit Context	Key Implications	References
DA Receptors	Expression of D1/D5, D2, D4; astrocytic D1/D5 required for non-Hebbian LTP; cortical laminar gradients (layer I > deep).	Spinal nociceptive pathways; cortical apical dendritic zones.	Establish astrocytic DA receptors as causal regulators of plasticity and cognition.	[6],[23]
Receptor Heteromers	D2-OTR and A2A-D2-OTR complexes; regulate Ca²⁺ and glutamate release; integrate DA, adenosine, oxytocin.	Striatal astrocytes.	Provide multimodal integration; implicated in reward learning and habit pathology.	[24]–[27]
Gliotransmission	DA-induced glutamate release; lowered threshold for Ca²⁺ waves; DA-glutamate-astrocyte loop.	Spinal circuits (LTP induction); striatum (interneuron-driven DA release).	Astrocytes function upstream and downstream of DA, modulating plasticity and reinforcement.	[6],[10],[28],[29]
Uptake & Clearance	Buffering of adenosine/GABA; EAAT1/2-mediated glutamate clearance.	Striatal DA terminals; cortico-thalamic inputs.	Real-time gating of DA tone; substrate for glutamate-DA crosstalk.	[10],[30],[31]
Regional Specificity	Striatal astrocytes: soma-to-soma contacts with interneurons, enriched heteromers. Cortical astrocytes: D1R/D4R-rich in layer I.	Striatum: phasic DA release, motor/reward control. Cortex: working memory, attention.	Dysregulation linked to PD, addiction, compulsive habits; schizophrenia, ADHD.	[10],[23]

### 2.1. Astrocytic DA receptors: Distribution, signaling consequences, and heteromers

Astrocytes express multiple subtypes of DA receptors, and their activation exerts direct consequences on neural circuit dynamics [10],[23],[32]. Within nociceptive pathways of the dorsal horn, astrocytic D1/D5 receptor activity is indispensable for a form of non-Hebbian long-term potentiation (LTP), as astrocyte-specific receptor knockdown abolishes plasticity at primary afferent synapses and neuronal knockdown does not [6],[33]. This establishes astrocytic DA receptors as causal determinants of synaptic gain and broadens the locus of dopaminergic influence beyond neurons.

Cortical investigations reveal region- and layer-specific gradients of receptor expression, with superficial astrocytes in layer I exhibiting strong immunoreactivity for D1R and D4R, moderate levels of D5R, and lower expression of D2R, whereas deeper protoplasmic astrocytes display substantially reduced expression [6],[10],[23],[32],[34]. These laminar differences suggest that astrocytic networks near pyramidal apical dendrites are positioned as hubs for top-down cortical modulation of dopaminergic tone.

Beyond individual receptor subtypes, astrocytes form heteromeric receptor complexes that expand their computational repertoire. Assemblies such as D2-oxytocin receptor heteromers and higher-order A2A-D2-oxytocin receptor complexes have been identified in striatal astrocytic processes [24],[27],[35]. These complexes regulate intracellular Ca²⁺ signaling and glutamate release, creating receptor-receptor interactions (RRIs) that function as molecular logic gates for convergent neuromodulatory inputs. Through these RRIs, astrocytes integrate dopaminergic, adenosinergic, and oxytocinergic signals, highlighting their role as computational integrators of neuromodulation rather than passive relay stations [25],[26].

### 2.2. Astrocytic gliotransmission as an upstream and downstream regulator of DA

Astrocytic gliotransmission operates upstream of DA release and downstream at postsynaptic sites [6],[9],[28],[29]. In nociceptive networks, D1/D5 receptor activation in astrocytes drives non-Hebbian LTP at major afferent synapses, even under conditions of minimal postsynaptic activity, indicating that astrocytes can independently set thresholds for synaptic potentiation [6],[32]. This expands the classical framework of plasticity and positions astrocytes as active drivers of long-term information storage.

In the striatum, astrocytes modulate DA release indirectly by regulating extracellular adenosine and GABA, which in turn shape the excitability of cholinergic interneurons. In vivo imaging demonstrates that astrocytic depolarization can rapidly shift interneuron firing and thus sculpt DA release dynamics on subsecond timescales [10],[36]–[38]. This astrocyte-interneuron axis reframes astrocytes as fast regulators of dopaminergic output, functioning with temporal precision previously attributed exclusively to neurons.

Astrocytes also act as cross-modal integrators at excitatory-dopaminergic interfaces. DA lowers the threshold for glutamate-evoked Ca²⁺ waves in astrocytes, thereby amplifying and propagating intracellular signals [28],[34],[39]. This synergy creates a bidirectional dopamine-glutamate-astrocyte loop, enabling fine-tuning of excitatory integration and circuit output.

### 2.3. Astrocytic uptake and clearance in DA regulation

Astrocytes critically regulate neuromodulator tone through uptake and clearance mechanisms. In the striatum, astrocytic buffering of GABA and adenosine modulates the inhibitory control of dopaminergic terminals via GABAA/GABAB and A1 receptors. Transient depolarization or disruption of astrocytic buffering alters cholinergic interneuron excitability and DA release within hundreds of milliseconds, underscoring the role of astrocytes as real-time gatekeepers of dopaminergic signaling [10],[37],[40],[41].

Although in vivo evidence for direct astrocytic involvement of excitatory amino acid transporter 1 and 2 (EAAT1/2) transporters in DA regulation remains limited, single-cell and spatial transcriptomics reveal that astrocyte subtypes within dopamine-rich regions differentially express EAAT isoforms [30],[31]. This suggests that glutamate clearance capacity indirectly modulates dopaminergic excitability, particularly where glutamate spillover from cortical or thalamic inputs could influence dopaminergic neurons or striatal projection neurons. By serving as buffers between excitatory drive and dopaminergic responsiveness, astrocytic EAATs provide a mechanistic substrate for glutamate-DA crosstalk [42],[43].

Collectively, these insights establish astrocytic transporters and buffering systems as hidden regulators of DA tone, integrating GABAergic, adenosinergic, and glutamatergic dynamics into the dopaminergic system and expanding the framework of DA regulation beyond neuronal boundaries.

### 2.4. Astrocytic modulation of DA: Circuit-specific mechanisms in the striatum and cortex

Astrocytic modulation of DA signaling displays striking regional specificity, reflecting the anatomical and computational demands of distinct circuits. In the striatum, astrocytes form specialized soma-to-soma “satellite” configurations with cholinergic interneurons, enabling direct influence over interneuron excitability. These structural interactions, combined with astrocytic buffering of adenosine and GABA, enable astrocytes to regulate DA release with remarkable temporal precision on subsecond timescales [10],[35],[44]. Striatal astrocytes are further enriched with heteromeric receptor complexes, including A2A-D2-oxytocin receptor assemblies, which couple to Ca²⁺ dynamics and glutamate gliotransmission [24],[27],[45]. This molecular machinery equips striatal astrocytes with multi-channel mechanisms for tuning phasic DA release, thereby shaping reinforcement learning, reward prediction error signaling, and motor control. Dysregulation of these astrocytic processes has been implicated in maladaptive dopaminergic states underlying PD, substance use disorders, and compulsive habit formation.

In contrast, astrocytic contributions in the PFC are defined by the laminar organization of cortical circuits. Superficial astrocytes in the pial and layer I zones display strong expression of D1R and D4R, moderate levels of D5R, and weak D2R enrichment, placing them in strategic proximity to pyramidal neuron apical dendrites [23]. These astrocytes are positioned to shape the apical integration of long-range inputs and are thought to engage Ca²⁺-dominated signaling cascades rather than classical cAMP-mediated pathways [46]. This lamina-specific dopaminergic responsiveness implicates cortical astrocytes in regulating higher-order processes such as working memory, attentional control, and cognitive flexibility. Perturbation in these astrocytic mechanisms may contribute to psychiatric pathophysiology, including schizophrenia and attention-deficit/hyperactivity disorder. Collectively, these findings underscore that astrocytic modulation of DA is spatially and functionally specialized, operating through distinct cellular principles in subcortical versus cortical territories [23],[32],[34].

### 2.5. Technological and translational advances enabling astrocyte-specific modulation of DA circuits

The recognition of astrocytes as active and temporally precise regulators of dopaminergic signaling has been driven by converging methodological advances that integrate genetic specificity, optical resolution, and molecular mapping. Together, these approaches have transformed astrocytes from conceptual modulators into experimentally tractable and therapeutically relevant components of DA circuits [6],[23],[32],[47].

Genetic strategies have provided the most compelling causal evidence for astrocytic control of dopamine-dependent plasticity. Conditional knockouts and astrocyte-specific receptor silencing demonstrate that elimination of D1/D5 receptors selectively in astrocytes, but not neurons, abolishes non-Hebbian long-term potentiation in nociceptive pathways, establishing astrocytic DA signaling as indispensable for circuit-level plasticity [6],[23],[32],[34],[48]. These findings decisively move astrocytes upstream of synaptic modification rules, rather than positioning them as secondary or permissive elements.

Optical technologies have further reshaped this framework by enabling real-time measurement of DA dynamics alongside astrocytic activity in vivo. Genetically encoded DA sensors, such as a G-protein-coupled receptor activation-based dopamine (GRAB-DA) sensor, combined with astrocyte-targeted calcium indicators (e.g., GCaMP), reveal astrocyte-interneuron interactions that regulate DA release with millisecond precision [6],[10],[33],[34],[49]. These observations overturn the traditional view of astrocytes as slow homeostatic buffers and instead position them as rapid, temporally precise integrators capable of sculpting neuromodulatory output on behaviorally relevant timescales.

At the molecular scale, advances in proximity ligation assays, super-resolution microscopy, and spatial transcriptomics have uncovered a previously unappreciated layer of astrocytic complexity. These approaches have delineated the subcellular organization of astrocytic receptor heteromers, including A2A-D2-oxytocin receptor assemblies localized to striatal astrocytic membranes, where they regulate Ca²⁺ signaling and gliotransmitter release [6],[9],[24],[27],[37]. Such receptor-receptor interactions provide a structural and biochemical framework for understanding how astrocytes integrate dopaminergic, adenosinergic, and neuropeptidergic signals, functioning as molecular logic gates that confer context sensitivity to astrocytic responses.

**Figure 1.**
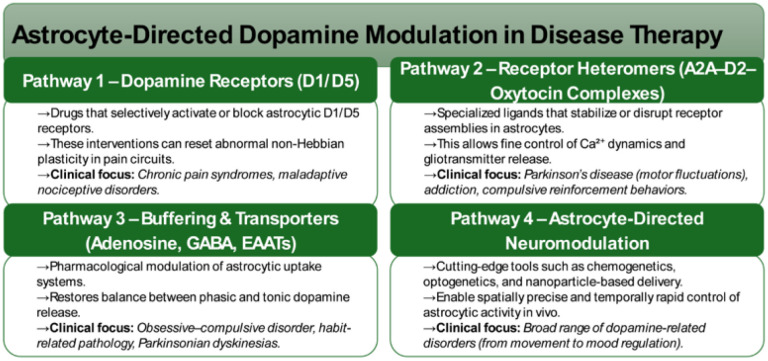
Astrocyte-targeted therapeutic pathways for modulating DA circuits in health and disease.

Importantly, these technological advances carry direct translational implications. Unlike traditional neuron-centric dopaminergic therapies, astrocyte-centered strategies offer opportunities for circuit-specific and state-dependent modulation of DA tone (Figure 1). Selective targeting of astrocytic D1/D5 receptors represents one potential therapeutic avenue, enabling normalization of maladaptive non-Hebbian plasticity in nociceptive and stress-related circuits and extending dopaminergic interventions beyond canonical neuronal targets [6],[9],[50]–[52].

Another promising strategy involves pharmacological manipulation of astrocytic receptor heteromeric complexes. The identification of A2A-D2-oxytocin receptor assemblies highlights novel druggable interfaces through which Ca²⁺ dynamics and gliotransmission can be fine-tuned with greater specificity than single-receptor approaches. Such strategies are particularly relevant to PD, where aberrant adenosine-DA interactions contribute to motor fluctuations, as well as to compulsive and addictive disorders characterized by maladaptive reinforcement learning [16],[24],[35],[45],[53].

Astrocytic buffering of adenosine and GABA constitutes an additional therapeutic axis. Modulating astrocytic transporter and uptake systems may restore the balance between phasic and tonic DA release, with potential applications in Parkinsonian dyskinesias, obsessive-compulsive disorder, and habit pathology [10],[35],[45],[54]. Beyond pharmacological interventions, astrocyte-directed neuromodulation technologies, including chemogenetic, optogenetic, and nanoparticle-based approaches, are emerging as powerful tools for achieving temporally precise and spatially restricted control of astrocytic activity. These platforms not only provide mechanistic insight but also establish astrocytes as viable therapeutic entry points rather than passive bystanders.

Despite these advances, important gaps remain. The direct contribution of astrocytes to DA transporter regulation is unresolved, and the full repertoire of gliotransmitters released under dopaminergic control remains incompletely defined [27],[32],[55],[56]. Moreover, astrocytic DA signaling exhibits marked regional and state-dependent variability, and its causal contribution to higher-order cognition and psychiatric phenotypes is only beginning to be explored. Addressing these challenges will require systematic integration of real-time DA sensing with astrocyte-specific perturbations during behavior, alongside causal testing in prefrontal and limbic circuits relevant to schizophrenia, attention-deficit/hyperactivity disorder, and mood disorders.

Collectively, the convergence of genetic, optical, and molecular technologies has repositioned astrocytes as central, experimentally accessible regulators of dopaminergic circuits. By aligning mechanistic precision with translational potential, astrocyte-centered approaches support a paradigm shift toward glia-neuron co-modulation and substantially expand the therapeutic landscape for disorders rooted in dopaminergic dysfunction.

## 3. Microglial-DA crosstalk and neuroinflammation

Building on astrocyte-mediated regulation of dopamine, we next focus on microglial-dopamine crosstalk, with particular emphasis on neuroinflammatory mechanisms relevant to Parkinson's disease.

PD is a progressive neurodegenerative disorder defined by the selective vulnerability of dopaminergic neurons in the substantia nigra pars compacta and the resulting loss of striatal dopamine. Clinically, PD presents with motor features such as bradykinesia, rigidity, and tremor, alongside prominent non-motor symptoms, including depression, fatigue, sleep disturbances, and cognitive impairment. Importantly, converging pathological and imaging evidence indicates that neuroinflammatory and glial alterations emerge early in disease progression, often preceding overt dopaminergic neuron loss [57],[58].

A central molecular hallmark of PD is the misfolding and aggregation of α-synuclein, a presynaptic protein involved in synaptic vesicle trafficking and neurotransmitter release. Under pathological conditions, α-synuclein forms oligomeric and fibrillar assemblies that accumulate within neurons and propagate across connected circuits. These aggregated species act as potent immune stimuli, engaging pattern-recognition receptors on microglia, activating inflammasome pathways, and promoting cytokine release. As such, α-synuclein serves as a key molecular bridge linking dopaminergic stress to microglial activation and neuroinflammation [59],[60].

Microglia are increasingly recognized as critical modulators of dopaminergic physiology. Beyond their classical roles in surveillance and synaptic pruning, microglia sense DA through receptor-mediated pathways, translate these signals into metabolic and inflammasome programs, and release cytokines that shape dopaminergic neuronal viability (Table 2). These bidirectional interactions are now implicated in prodromal PD, systemic immune comorbidities, such as HIV, and stress-related psychiatric syndromes, positioning microglia as context-dependent amplifiers, or brakes, of DA biology [13],[61].

**Table 2.** Microglial regulation of DA signaling and neuroinflammation.

Mechanistic Domain	Mechanistic Description	Representative Evidence	Pathological Relevance
Receptor-mediated signaling	Microglia express DRD1-DRD4 (DRD2/DRD4 enriched). DRD2 signaling suppresses inflammasome activity; DRD1 enhances pro-inflammatory cascades. Balance dictates outcome.	Transcriptomic and proteomic validation [3]–[6]; functional suppression or activation of NLRP3 inflammasome by DA [7],[8].	DA acts as context-dependent brake (via DRD2) or amplifier (via DRD1) of neuroinflammation.
Inflammatory polarization	DA tunes microglial states: DRD2 biases toward reparative, anti-inflammatory programs; DRD1 predominance amplifies IL-1β and NF-κB signaling.	Transcriptomic induction of cytokine and inflammasome genes in DA-exposed microglia [1],[9]–[11].	Determines switch between neuroprotection and neurotoxicity; central in chronic immune stress and HIV.
Stress and metabolic priming	Chronic stress alters lipid metabolism, mitochondrial respiration, and redox balance, lowering microglial activation thresholds. DA stimulation more readily triggers inflammasome activity.	GRAB-DA recordings in stress paradigms [12],[13]; PET studies in prodromal PD showing immune activation preceding DA decline [14],[15].	Exaggerated immune reactivity; heightened vulnerability to depression, anhedonia, and neurodegenerative progression.
Proteinopathy and toxic metabolites	α-Synuclein activates TLR2/TLR4-NF-κB-NLRP3 axis; DA-derived metabolites (DOPAL, quinones) induce oxidative stress and toxic adduct formation.	α-synucleinopathy models show cytokine release and pathology propagation [19]–[22]; DOPAL-α-synuclein adducts accelerate fibrillization [26]–[28].	Amplifies microglial pro-inflammatory bias; accelerates dopaminergic degeneration in PD.
Reciprocal immune-DA loops	Microglial cytokines (IL-1β, TNF-α, IL-6) suppress TH, blunt DA release, and disrupt dopamine transporter (DAT) trafficking. Systemic inflammation reprograms DA-microglia interactions.	Longitudinal PET studies in prodromal PD [29],[30]; HIV and systemic immune models show maladaptive DA-driven inflammasome activation [1].	Self-reinforcing cycle where inflammation impairs DA signaling and DA dysregulation further activates microglia.

It is important to note that the classical M1/M2 dichotomy of microglial activation is now widely regarded as an oversimplification. Contemporary single-cell, spatial transcriptomic, and proteomic studies demonstrate that microglial states exist along a dynamic, multidimensional continuum shaped by developmental stage, regional identity, immune context, and disease state [62],[63]. Accordingly, throughout this review, terms such as “pro-inflammatory” or “reparative” microglial states are used as functional descriptors rather than rigid polarization categories and do not imply discrete or stable cell identities.

### 3.1. DA receptor expression and signaling in microglia

Large-scale transcriptomic and proteomic studies confirm that microglia possess a selective repertoire of DA receptors. Across human and rodent systems, DRD2 and DRD4 are consistently enriched, DRD1 and DRD3 are expressed at moderate levels, and DRD5 expression is minimal [64],[65]. Crucially, these are not merely transcriptional traces but functional proteins capable of initiating intracellular cascades. DRD2 engagement couples to Gi/o signaling pathways, reducing cAMP levels and influencing downstream inflammasome activity, while DRD1 couples to Gs-cAMP-PKA signaling, thereby promoting opposing effects [66],[67].

Functional assays in human primary microglia and immortalized lines reveal that DA signaling through DRD1 and DRD2 can suppress NLRP3 inflammasome activation, reducing IL-1β release under canonical (LPS/ATP), non-canonical (caspase-11), and proteinopathy-associated (α-synuclein) challenges [68],[69]. This positions DA as an endogenous checkpoint regulator, constraining excessive immune activation and preventing runaway neuroinflammation. In this framework, DA emerges not only as a neuromodulator of synaptic transmission but also as an immunomodulatory signal critical for maintaining homeostatic balance.

Importantly, these effects are not uniform: Dopamine exerts dual and sometimes opposing influences on microglial state depending on receptor subtype engagement, receptor balance, immune context, and disease state, a principle that underlies the apparent heterogeneity of dopaminergic immune regulations discussed below.

### 3.2. DA control of microglial polarization and inflammatory tone

Building on the receptor-specific signaling framework outlined above, dopamine regulation of microglial polarization is inherently bidirectional, with distinct receptor subtype dominance determining whether dopaminergic signaling constrains or amplifies inflammatory programs.

Microglia operate along a polarization spectrum spanning pro-inflammatory and reparative functional states rather than discrete M1/M2 categories. DA signaling exerts a powerful influence over this spectrum, but its effects are highly dependent on receptor balance, local immune context, and disease state. Under physiological conditions, DRD1/DRD2 activation promotes anti-inflammatory programs: Attenuating NLRP3 activation, reducing IL-1β production, and upregulating reparative gene networks. Such actions are consistent with neuroprotective phenotypes observed in preclinical models of delirium and Parkinsonian pathology [70],[71].

However, the ratio of DRD1-like to DRD2-like signaling appears to function as a regulatory switch. When DRD1 activity predominates, DA stimulation paradoxically amplifies IL-1β transcription, enhances inflammasome activity, and drives sustained pro-inflammatory responses, particularly under chronic immune challenge such as HIV infection [13]. In contrast, DRD2 signaling exerts counter-regulatory effects, restraining this pro-inflammatory cascade. Transcriptomic profiling of dopamine-exposed microglia further supports this duality, revealing coordinated induction of IL-1β pathway genes, inflammasome adaptors, and NF-κB targets under conditions of receptor imbalance [12],[13],[71].

Together, these findings establish DA as a context-sensitive modulator of microglial state, capable of functioning either as an anti-inflammatory brake or a pro-inflammatory accelerator. The outcome is determined not by DA alone, but by the dynamic balance of receptor subtype activation, immune context, and cellular state: A principle that has profound implications for neurodegeneration, neuroinflammation, and psychiatric disease.

### 3.3. Stress, trauma, and depression: Priming the microglial-DA axis

Although direct investigations of dopamine-microglia interactions in stress-related and depressive disorders remain limited, converging mechanistic evidence implicates microglial metabolic priming as a central vulnerability factor. Chronic stress reshapes microglial physiology by altering lipid metabolism, mitochondrial respiration, and redox balance, thereby lowering the threshold for immune activation. Under these conditions, dopaminergic stimulation more readily engages inflammasome pathways, producing exaggerated pro-inflammatory responses in stressed neural circuits [72],[73]. This metabolic reprogramming effectively converts DA from a homeostatic signal into a trigger for maladaptive immune amplification.

Clinical neuroimaging provides convergent evidence. In idiopathic Rapid Eye Movement (REM) sleep behavior disorder, widely regarded as a prodromal stage of PD, translocator protein-positron emission tomography (TSPO-PET) imaging reveals heightened microglial activation in the substantia nigra and basal ganglia. These immune changes correlate with reduced DA transporter binding and diminished tyrosine hydroxylase activity, suggesting that immune activation may precede, or at minimum parallel, dopaminergic decline [74],[75]. Such findings lend support to a feed-forward model in which microglial priming accelerates the trajectory from stress-linked vulnerability to neurodegenerative pathology.

Experimental models further illuminate the heterogeneity of stress-responsive microglia. Distinct inflammatory microglial subtypes have been identified in α-synucleinopathy and tauopathy contexts, enriched for gene signatures linked to lipid metabolism, immune cell trafficking, and inflammasome activation [76],[77]. While direct characterization of DA receptor expression within these subtypes is lacking, their transcriptional profiles strongly suggest increased sensitivity to dopaminergic modulation. This framework provides a mechanistic bridge between stress-induced microglial priming and the heightened susceptibility to dopamine-driven inflammatory cascades, with implications extending across psychiatric and neurodegenerative disorders.

### 3.4. Microglial contributions to dopaminergic vulnerability in PD

Importantly, the relationship between microglial activation and dopaminergic degeneration in PD is not strictly unidirectional. While substantial evidence demonstrates that activated microglia can exacerbate dopaminergic vulnerability through inflammatory, metabolic, and oxidative mechanisms, dopaminergic neuron stress and degeneration can themselves initiate and sustain microglial activation. Data, therefore, support a bidirectional, feed-forward model in which microglial inflammatory programs and dopaminergic dysfunction mutually reinforce one another, with the relative contribution of each component varying across disease stage, brain region, and genetic or environmental context [78],[79].

The contribution of microglia to dopaminergic degeneration in PD is recognized as the outcome of converging processes involving protein aggregation, mitochondrial dysfunction, and dysregulated DA catabolism [80]. Aggregated α-synuclein interacts with pattern recognition receptors, such as TLR2 and TLR4 on microglia, initiating NF-κB-NLRP3 inflammasome signaling and driving the release of pro-inflammatory cytokines including IL-1β, (tumor necrosis factor-α) TNF-α, and IL-6 [81],[82]. This inflammatory cascade not only accelerates neuronal injury but also facilitates the cell-to-cell propagation of α-synuclein pathology. Post-mortem analyses consistently reveal microgliosis within the substantia nigra and striatum, while in vivo PET imaging with TSPO ligands demonstrates widespread microglial activation in PD, closely correlating with motor severity and dopaminergic loss [83],[84].

Mitochondrial dysfunction further amplifies this inflammatory milieu. Damage to dopaminergic neurons and microglia generate excessive reactive oxygen species and releases mitochondrial DNA, cardiolipin, and other danger-associated molecular patterns, sustaining chronic inflammasome activity [85],[86]. Transcriptomic profiling of inflammatory microglial states in α-synucleinopathy and tauopathy models highlights profound alterations in lipid metabolism and mitochondrial respiration, features that bias microglia toward pro-inflammatory polarization while diminishing their neuroprotective capacity [87].

In parallel, DA metabolism contributes toxic amplifiers of neurodegeneration. The reactive metabolite 3,4-dihydroxyphenylacetaldehyde (DOPAL) forms adducts with α-synuclein, enhances fibrillization, and exerts direct cytotoxic effects on dopaminergic neurons [88]. Impairments in detoxification pathways, such as aldehyde dehydrogenase deficiency, enable the accumulation of dopamine-derived quinones and aldehydes, which impose oxidative and electrophilic stress that further bias microglia toward pro-inflammatory states [89]. Integrative analyses now emphasize how this metabolite-driven toxicity intersects with genetic susceptibility loci, including LRRK2 and GBA, to potentiate microglial activation and accelerate the trajectory of neurodegeneration [90].

Taken together, these findings indicate that dopaminergic degeneration in PD emerges from reciprocal interactions, in which aggregated α-synuclein, mitochondrial distress, and reactive DA metabolites converge on shared inflammasome and oxidative pathways. This convergence not only heightens dopaminergic vulnerability but also perpetuates a self-reinforcing cycle of neuroinflammation and neuronal loss, underscoring the critical role of microglial mechanisms as amplifiers and modulators of disease progression.

### 3.5. Reciprocal immune-DA feedback loops

Microglia and dopaminergic neurons are engaged in reciprocal feedback circuits that, when dysregulated, amplify pathology across neurodegenerative and psychiatric conditions. Activated microglia release cytokines, such as IL-1β, TNF-α, and IL-6, which downregulate tyrosine hydroxylase expression, blunt DA release, and disrupt DA transporter trafficking [91]. These immune-mediated suppressive effects on DA synthesis are consistent with findings from preclinical toxin models as well as human neuroimaging. Longitudinal PET investigations in prodromal cohorts, such as idiopathic REM sleep behavior disorder, reveal that heightened microglial activation precedes measurable striatal dopaminergic decline, suggesting that immune activity is not a secondary consequence but an early driver of nigrostriatal dysfunction [92].

Peripheral immune comorbidities further recalibrate dopamine-microglia interactions. In states of chronic systemic inflammation or infection, such as HIV, DA signaling assumes a maladaptive profile. Human macrophage and microglial studies demonstrate that immune challenge amplifies dopamine-induced inflammasome activation and IL-1β release, an effect dependent on the relative balance of DRD1-like versus DRD2-like receptors [13]. This finding underscores how systemic immune states can reprogram microglial DA sensitivity, potentially explaining the heightened vulnerability to neuroinflammation in patients receiving dopaminergic therapies or psychostimulants in the context of comorbid infections. Thus, DA not only modulates microglial state but is itself reshaped by immune tone, creating a self-reinforcing loop wherein inflammation drives dopaminergic dysfunction and impaired DA signaling further potentiates microglial activation.

Despite these advances, several critical uncertainties remain. Most in vitro studies expose microglia to supraphysiologic DA concentrations, leaving unresolved how cells respond to physiologically relevant tonic versus phasic release across striatal and mesocortical circuits [12]. Conflicting reports of dopamine's anti-inflammatory versus pro-inflammatory roles likely reflect differences in receptor subtype balance, microglial activation state, and systemic immune comorbidities, highlighting the need for receptor-specific in vivo perturbations through conditional knockouts or chemogenetics [70]. The impact of DA metabolites, particularly reactive species such as DOPAL and DA quinones, remains underexplored: While cytotoxic to neurons, their direct influence on microglial receptor signaling, redox balance, and inflammasome priming has not been systematically defined [93].

Defining whether microglia sense and respond differently to DA versus its metabolites will be critical to understanding disease-specific pathophysiology. Regional and temporal specificity further complicate interpretation. Microglial responses to DA likely differ between substantia nigra, ventral tegmental area, striatum, and PFC, and these trajectories may shift across disease stages [10],[12]. Determining when and where microglia transitions from protective to deleterious roles will require harmonized approaches that integrate longitudinal TSPO-PET imaging, cerebrospinal fluid cytokine readouts, and single-cell transcriptomic profiling. Finally, stress and depression represent underexplored modulators of this axis. While chronic stress is known to prime microglia toward pro-inflammatory states, direct evidence linking DA receptor remodeling in microglia to stress-induced anhedonia or motivational deficits remains limited, underscoring the need for targeted molecular and imaging studies in affective disorders [12].

Collectively, these gaps highlight the urgent need for a mechanistic framework that accounts for dose, receptor balance, metabolite exposure, regional context, and systemic immune status. Only through such multidimensional integration can the field fully delineate how microglial-DA feedback loops shape vulnerability to neuroinflammation, neurodegeneration, and psychiatric disease.

## 4. Astrocyte-microglia interactions in DA circuits

Because astrocytes and microglia rarely act in isolation, we next examine how their reciprocal interactions form integrated regulatory networks that shape dopaminergic circuit stability and vulnerability.

### 4.1. From tripartite to network synapses: Expanding the framework of dopaminergic circuit regulation

The classical tripartite synapse, originally described as the interplay between pre- and postsynaptic neurons with perisynaptic astrocytic processes, has been substantially revised. Accumulating evidence supports an expanded quadripartite model in which microglia act as active and contact-competent partners that influence synapse formation, elimination, and efficacy. Complement-dependent microglial pruning, first identified in development, is recognized as a lifelong mechanism that contributes to activity-dependent remodeling of dopaminergic circuits in the striatum and PFC [94]–[96]. Extending beyond this framework, the concept of the “network synapse” has emerged, integrating astrocytic endfeet, perivascular microglia, endothelial cells, and pericytes. This expanded view underscores the role of vascular and immune influences ranging from metabolic alterations to systemic inflammatory states in modulating dopaminergic plasticity [97],[98]. By situating DA synapses within a glial-vascular-immune ecosystem, the field now conceptualizes dopaminergic signaling as dynamically embedded in systemic physiology and inflammatory tone.

### 4.2. Molecular axes of astrocyte-microglia crosstalk in DA regulation

Astrocytes and microglia engage in reciprocal signaling loops that directly influence dopaminergic circuits. Cytokine- and complement-mediated communication is a central axis of this dialogue: Microglia-derived IL-1β, TNF-α, and complement proteins drive astrocytes toward phagocytic and pro-inflammatory states, while astrocytic secretion of IL-6 and TGF-β conditions microglial polarization and establishes thresholds for synaptic engulfment (Table 3) [99]–[102]. Moreover, single-cell and spatial transcriptomic analyses demonstrate that astrocytic reactivity cannot be reduced to a binary A1/A2 paradigm but instead reflects heterogeneous and region-specific states that variably shape dopaminergic vulnerability [103],[104].

Other communication channels reinforce this interglial interplay. Purinergic signaling synchronizes DA release with immune surveillance, as astrocytic regulation of adenosine and GABA tunes cholinergic interneurons, the principal gatekeepers of striatal DA release, while microglial purinergic receptor activity regulates motility and surveillance behaviors [105]–[107].

Chemokine-based signaling, particularly the CX3CL1-CX3CR1 axis, further coordinates astrocytic coverage with microglial process engagement, with shifts in fractalkine sources during pathology linked to maladaptive microglial activation and dopaminergic vulnerability [108],[109]. Additionally, extracellular vesicles (EVs) released by astrocytes, microglia, and neurons carry cytokines, miRNAs, and metabolic cargo that function as biomarkers of glial state and as effectors of inflammatory propagation and synaptic remodeling [110]–[112].

Taken together, these reciprocal pathways form feed-forward and feedback networks in which astrocytic gliotransmission and microglial activation converge to regulate DA release dynamics, receptor composition, and long-term plasticity. In this framework, astrocyte-microglia interactions do not serve as a background modulatory system but constitute a computational layer of dopaminergic circuitry, integrating neuromodulatory, immune, and vascular signals to shape resilience and disease vulnerability [113].

**Table 3.** Mechanistic and translational dimensions of astrocyte-microglia interactions in DA circuits.

Thematic Focus	Core Concept	Mechanistic Axes	DA Circuit Effects	Clinical/Translational Relevance
Molecular Crosstalk	Reciprocal astrocyte-microglia signaling	- Cytokines: IL-1β, TNF-α ↔ IL-6, TGF-β - Complement: synaptic tagging & pruning - Purinergic: ATP/adenosine, P2Y12 - Chemokine: CX3CL1-CX3CR1 - Extracellular vesicles (EVs): cytokines, miRNAs, α-syn cargo	- Sets thresholds for engulfment - Tunes release dynamics & receptor composition - Couples immune surveillance to DA signaling	- Aberrant loops drive neuroinflammation & maladaptive pruning - EVs as biomarkers and therapeutic carriers
Spatial & Single-Cell Landscapes	Glial heterogeneity in DA hubs	- Oxidative-metabolic astrocyte subtypes (antioxidant, mitochondrial) - Immune-primed microglial clusters (ISGs, inflammasomes, lipid metabolism) - Pathological niches: astrocytes + microglia + infiltrating T cells	- Defines “glial neighborhoods” embedded in D1/D2 ensembles - Orchestrates reinforcement learning & motivational plasticity	- PD: emergence of glial-immune niches - Astrocytic diversity in health vs. microglial expansion in pathology - Biomarkers for selective vulnerability
Mechanisms of Plasticity	Glia as regulators of dopaminergic rules	- Astrocytic adenosine/GABA → cholinergic interneuron excitability - Astrocytic GPCR heteromers (A2A-D2, A2A-OTR, D2-OTR) - Microglial complement-mediated pruning - Vascular-metabolic coupling (ROS buffering, BBB integrity)	- Determines the phasic/tonic DA balance - Defines thresholds of corticostriatal plasticity - Shapes reinforcement learning algorithms	- Disrupted metabolic support/vascular surveillance drives degeneration - Aberrant pruning and heteromer logic linked to PD, stress, addiction
Disease Microenvironments	Glia-centric niches of vulnerability	- Lipid- and inflammasome-enriched microglia + oxidative-stress astrocytes - Fractalkine signaling shift (neuronal → endothelial) - Glia-derived EVs propagate α-syn & inflammatory cargo	- Maladaptive niches amplify dopaminergic vulnerability - Remodels mesocorticolimbic circuits under chronic stress	- PD: neuroinflammation + metabolic collapse - Stress: impaired DA release, motivational deficits (anhedonia, apathy) - Engineered EVs as therapeutic vectors
Humanized In Vitro Models	iPSC-derived tri-cultures for mechanistic precision	- Recapitulate IL-1β/TNF-α loops, complement, purinergic cascades - Readouts: synapse density, excitability, EV cargo	- Enable controlled dissection of astrocyte-microglia-neuron interactions - Define causal modules in DA regulation	- Preclinical pipeline for drug discovery - Complement inhibition and purinergic modulation normalize excitability

### 4.3. Spatial and single-cell landscapes of astrocytic and microglial heterogeneity in dopaminergic circuits

Advances in single-cell and spatial multi-omics have provided unprecedented resolution of astrocytic and microglial diversity in dopaminergic hubs. In the human substantia nigra, single-cell and single-nucleus atlases consistently identify oxidative-metabolic astrocyte subtypes enriched for genes involved in antioxidant defense, mitochondrial regulation, and lactate shuttle pathways (Table 3) [114]. In parallel, immune-primed microglial clusters have been mapped, characterized by interferon-stimulated genes, inflammasome components, and lipid metabolism programs, suggesting that local glial heterogeneity is a critical determinant of dopaminergic resilience versus vulnerability [115].

In PD, integrated single-nucleus and spatial multi-omics analyses reveal the emergence of glial-immune niches defined by close apposition of reactive astrocytes, inflammatory microglia, and infiltrating T cells to degenerating DA neurons [116]. These niches exhibit coordinated transcriptomic programs involving antigen presentation, cytokine signaling, and oxidative stress responses, supporting the concept that selective DA neuron loss arises not from neuron-autonomous processes but from interglial-immune coupling within the microenvironment.

Spatial mapping in experimental models has further delineated inflammatory gradients across the substantia nigra, showing that astrocytic and microglial activation states are spatially orchestrated around DA territories. These gradients correlate with mitochondrial dysfunction and synaptic attrition in nearby neurons, highlighting the regional coordination of glial-neural interactions during degeneration [117],[118]. In the striatum, longitudinal single-cell analyses across development and aging demonstrate protracted, lineage-specific transcriptional programs in astrocytes and microglia that shape the rules of DA receptor-specific plasticity. These studies define dynamic “glial neighborhoods” that interact with D1- and D2-receptor ensembles, embedding reinforcement learning mechanisms within a glial context [119],[120].

An emerging integrative theme across datasets is that astrocytic diversity predominates under homeostatic conditions, while microglial heterogeneity expands dramatically under disease or inflammatory stress. This reciprocal choreography, astrocytic specialization in health and microglial diversification in pathology, recalibrates dopaminergic microenvironments, with direct implications for neuronal resilience and selective vulnerability [116],[121].

### 4.4. Interglial mechanisms as architects of dopaminergic signaling and plasticity

The molecular and cellular heterogeneity revealed by single-cell approaches has direct consequences for the rules of DA signaling and plasticity. Astrocytic regulation of extracellular adenosine and GABA determines the excitability of striatal cholinergic interneurons, which in turn set the timing and amplitude of sub-second DA release. Perturbations of this axis can flip the polarity of DA receptor modulation, altering interneuron firing and DA availability [10],[105]. In addition, astrocytic G-protein-coupled receptor heteromers, such as A2A-D2, A2A-oxytocin receptor, and D2-oxytocin receptor complexes, act as molecular logic nodes that fine-tune release probability and plasticity thresholds, conferring context-dependent precision to DA signaling (Table 3) [27],[45].

Microglial complement-dependent pruning further contributes to circuit refinement by selectively sculpting dopaminergic synapses. In coordination with astrocytic “eat-me” and “keep-me” signals, this process determines the balance between D1- and D2-biased ensembles and thereby the rules of corticostriatal plasticity and reinforcement learning [95],[122]. Importantly, complement-tagged pruning persists into adulthood, implying that adaptive and maladaptive remodeling of DA circuits under stress, drug exposure, or disease arises from ongoing glial surveillance [123],[124].

Metabolic and vascular coupling provides a third axis of regulation. Astrocytic endfeet and perivascular microglia integrate glucose and lactate shuttling, reactive oxygen species buffering, and blood-brain barrier integrity. Under mitochondrial stress or α-synuclein accumulation, these glial-vascular checkpoints become decisive determinants of DA neuron survival [116],[125]. Disruption of astrocytic metabolic support or microglial vascular surveillance precipitates neuronal loss in experimental models and human PD tissue, underscoring the centrality of glial metabolism in dopaminergic neurodegeneration [74],[126],[127].

Together, these mechanisms demonstrate that astrocyte-microglia interactions not only modulate DA circuits but define the computational rules of plasticity. By setting the balance between phasic and tonic release, determining synaptic selection and remodeling, and calibrating metabolic resilience, interglial signaling emerges as a primary architect of dopaminergic circuit function. Evidence thus reframes plasticity as a glia-dependent property of the striatum and midbrain, with astrocytic and microglial crosstalk positioned upstream of dopaminergic computation.

### 4.5. Interglial microenvironments as determinants of dopaminergic degeneration and motivational dysfunction

Single-nucleus and spatial multi-omics have revealed that dopaminergic degeneration in PD unfolds within glia-centric niches of vulnerability. In the substantia nigra, microglial populations enriched in lipid metabolism, interferon-response, and inflammasome-related transcripts colocalize with astrocytic states characterized by oxidative stress, mitochondrial dysregulation, and disrupted glutamate handling (Table 3) [116]. These convergent programs suggest that dopaminergic cell loss is not solely the consequence of intrinsic neuronal fragility but reflects maladaptive microenvironments in which astrocytic and microglial states synergistically amplify vulnerability.

Meta-analyses of single-nucleus RNA-seq datasets reinforce this perspective, consistently highlighting microglial neuroinflammation and astrocytic metabolic/trophic dysregulation as recurrent axes of disease progression [128]. A striking example of this interglial remodeling is the observed shift in fractalkine (CX3CL1) signaling. Neuronal CX3CL1 expression diminishes in PD, while endothelial expression increases, thereby disrupting CX3CR1-mediated homeostatic signaling and biasing microglia toward maladaptive surveillance and heightened inflammatory activity [109].

EVs have also emerged as central mediators of PD pathophysiology. Glia-derived EVs carry α-synuclein, inflammatory factors, and microRNAs that propagate pathology across dopaminergic territories [112]. Furthermore, engineered EVs are being investigated as therapeutic carriers for anti-inflammatory or pro-metabolic cargo, offering a translational pathway for restoring dopaminergic resilience [129].

Chronic stress and motivational pathology reveal parallel themes of interglial remodeling. Sustained inflammatory states whether systemic or centrally generated retune astrocyte-microglia cytokine and purinergic signaling loops within mesocorticolimbic circuits. This remodeling reduces phasic DA signaling and contributes to motivational deficits, including anergia, anhedonia, and apathy [130],[131]. Transcriptomic studies show that prolonged stress expands the repertoire of reactive astrocytic and microglial states, many of which impair DA release and receptor-specific plasticity in the ventral striatum and PFC [132]. These findings support a mechanistic framework in which glial heterogeneity mediates the link between inflammatory tone, dopaminergic dysfunction, and stress-related psychiatric comorbidities such as depression and apathy.

### 4.6. In vitro humanized models of interglial signaling in DA circuits: Toward mechanistic precision and translation

Progress in human iPSC-derived co-culture and tri-culture systems has enabled systematic dissection of astrocyte-microglia-neuron interactions under controlled conditions. These platforms faithfully recapitulate key inflammatory axes, including IL-1β/TNF-α loops, complement signaling, and purinergic cascades, while providing real-time readouts of dopaminergic endpoints such as synapse density, electrophysiological excitability, and extracellular vesicle cargo [133]. Critically, they bridge the gap between reductionist assays and in vivo complexity, offering scalable platforms for mechanistic perturbation and therapeutic discovery.

Interventions in these models have demonstrated translational promise. For example, inhibition of complement pathways or modulation of P2Y12 signaling in tri-cultures normalizes dopaminergic neuron excitability, underscoring the causal role of specific interglial modules (Table 3) [134]. These advances highlight the utility of next-generation co-culture systems, not only for mechanistic dissection but also as preclinical pipelines for identifying candidate therapies.

### 4.7. Computational neuroscience of glial-DA interactions: From molecular states to circuit-level algorithms

Theoretical neuroscience has begun to incorporate astrocytic and microglial states into models of DA circuits, reframing glia as computational rather than modulatory elements. Contemporary frameworks treat glial calcium dynamics, receptor occupancy, metabolic buffering, and gliotransmission as slow variables that define neuronal gain control, eligibility traces, and synaptic plasticity thresholds [135]. This reconceptualization positions interglial signaling as a “plasticity thermostat” that sets the computational boundaries within which dopaminergic teaching signals operate [19],[136].

Embedding glial diversity into reinforcement-learning and network models enables simulations that predict how inflammatory or metabolic perturbations are biased toward behavioral strategies; for example, shifting exploration-exploitation balance, altering habit consolidation, or impairing motivational drive. Such integrative approaches generate testable hypotheses, linking molecular glial states to system-level dysfunction across PD, depression, and addiction.

Advancing this agenda requires a set of defined priorities. Foremost is the need for causal mapping, which demands the simultaneous resolution of dopaminergic dynamics and glial states in vivo. This will require the integration of fast-scan cyclic voltammetry or comparable DA sensors with glial-specific optical reporters and spatial transcriptomic approaches in behaving animals, thereby enabling a direct link between interglial signaling modules and dopaminergic output. Equally critical is the delineation of region-specific motifs, achieved by systematically comparing interglial signatures across the substantia nigra, ventral tegmental area, striatum, and PFC using integrated single-cell, spatial, and proteogenomic atlases. Parallel translational pipelines should focus on therapeutic logic, rigorously testing targeted ligands for astrocytic GPCR heteromers, modulators of purinergic cascades, and engineered (EVs) in human iPSC-derived tri-culture systems and in vivo models. Finally, computational integration will be indispensable. Embedding glial states into decision-theoretic and reinforcement-learning models will provide a framework to predict how inflammatory and metabolic set points reshape dopaminergic computations and downstream behavioral algorithms. By bridging molecular modules to system-level functions, these convergent strategies aim to construct a mechanistic continuum from interglial biology to the computational architecture of DA circuits.

## 5. Dopamine-glia interfaces in motivation and stress

Motivation and stress engage partially overlapping mesolimbic and mesocortical circuits, with DA signaling orchestrating key processes such as reward anticipation, effort allocation, reinforcement learning, and stress adaptation [137],[138]. While neurons have traditionally been viewed as the primary drivers of these processes, evidence demonstrates that astrocytes and microglia exert active, phase-specific, and circuit-localized control over DA dynamics. These glial mechanisms shape cue encoding, regulate strategy selection between goal-directed and habitual actions, and contribute to susceptibility or resilience in stress-induced anhedonia (Table 4). Importantly, the convergence of causal manipulation tools (optogenetics, chemogenetics) with phase-resolved monitoring approaches (GRAB-DA fiber photometry, calcium imaging) has enabled precise dissection of how glial activity governs anticipatory versus consummatory epochs of reward processing [139],[140].

**Table 4.** Astrocytic and microglial modulation of DA circuits in motivation and stress.

Glial Mechanism	Circuit/Region	Experimental Evidence	Behavioral/Clinical Outcome
Astrocytic ensembles encoding reward cues	Nucleus accumbens (NAc, posterior-ventral)	Activity-defined astrocytic ensembles recruited during cue-reward learning; optogenetic reactivation drives approach behavior [5],[6]	Astrocytes encode motivational salience; selective ensembles modulate cue-driven reward pursuit
Astrocytic regulation of strategy selection	External globus pallidus (GPe, indirect pathway)	Chemogenetic activation reduces habitual responding, enhances goal-directed actions; recruitment during action sequences [7],[8]	Balances motivational persistence with behavioral flexibility
Stress-sensitive astrocytic modulation of DA	NAc (chronic social stress model)	GRAB-DA fiber photometry shows attenuated anticipatory DA under stress; astrocytic glutamate/ATP/adenosine/D-serine implicated [9],[10]	Impaired effort allocation and reward learning; reduced motivational vigor
Microglial activation under chronic stress	NAc, PFC, VTA	Stress induces hypertrophy, immune gene upregulation, synaptic remodeling [11]; amplified with psychostimulants [12]	Impaired reward learning; vulnerability to anhedonia and addiction-like behaviors
Cytokine-DA coupling	Mesolimbic and mesocortical circuits	uCMS models show elevated IL-1β, TNF-α linked to anhedonia [13],[14]; clinical studies confirm microglial activation and inflammation in MDD [15],[16]	Cytokine-mediated suppression of DA signaling → reduced motivation
Microglial priming in early-life stress	Developmental NAc and mesolimbic circuits	ELS increases pro-inflammatory cytokines, disrupts HPA axis, blunts anticipatory reward [17],[18]; microglia regulate synaptogenesis during adolescence [19]	Lasting motivational vulnerability; reduced resilience to later stressors
Astrocytic gliotransmission and DA release control	NAc, GPe	Astrocytic glutamate, ATP, and D-serine release regulate MSNs and DA terminal excitability [5],[20]; GPe astrocytes bias flexible strategies [7]	Fine-tunes motivational encoding; shifts balance from habits to adaptive behaviors
Microglial cytokine release	NAc, PFC, VTA	IL-1β and TNF-α suppress DA synthesis, impair receptors, prune dendritic spines [21],[22]	Blunted reward anticipation; anhedonia
Developmental trajectory of glial-DA coupling	NAc during adolescence	Microglial regulation of synaptogenesis disrupted by ELS, immune stress, genetic risk [23]	Long-term alterations in connectivity → chronic motivational deficits
Real-time DA monitoring with GRAB-DA sensors	NAc, mesolimbic DA terminals	Photometry distinguishes anticipatory vs. consummatory DA; stress selectively attenuates anticipatory responses [24],[25]	Identifies anticipatory phase as window of vulnerability and therapeutic intervention
Behavioral assays linked to astrocytic manipulation	Operant paradigms (progressive ratio, outcome devaluation)	Chemogenetic GPe astrocyte activation biases goal-directed over habitual actions [7]; ensemble reactivation drives approach [5],[27]	Establishes causal role of astrocytes in motivational computation
Integrated immune-DA-behavior paradigms	CSS, uCMS, ELS models	Cytokine profiling + DA photometry map inflammation-DA-behavior triads [28],[29]	Clarifies convergence of neuroimmune signaling and motivational pathology

### 5.1. Astrocytic modulation of reward-seeking behavior and motivational states

A landmark advance has been the identification of activity-defined astrocytic ensembles within the posterior-ventral nucleus accumbens (NAc). Using a light-dependent transcriptional reporter, a sparsely distributed subset of astrocytes was shown to be selectively recruited during cue-reward learning [141]. Optogenetic reactivation of this ensemble alone without indiscriminate stimulation of the broader astrocytic population was sufficient to drive cue-motivated approach behavior, revealing an ensemble-level astrocytic code for motivational salience. These findings align with broader evidence that astrocytes exert input-specific, temporally precise neuromodulation rather than broad gain control, reframing them as active encoders of motivational signals [25].

Astrocytes in the external globus pallidus (GPe) play a regulatory role in motivational flexibility. Chemogenetic activation of GPe astrocytes has been shown to reduce habitual responding and enhance goal-directed actions in operant tasks [142]. Additionally, researchers have extended these findings, showing that GPe astrocytes are selectively recruited during reward-seeking action sequences and contribute to action-sequence refinement and strategic updating under repetitive conditioning paradigms [143]. Together, these observations highlight a circuit-level mechanism by which astrocytes influence striato-pallidal computations to tune the balance between motivational persistence and behavioral flexibility.

Stress paradigms emphasize the vulnerability of the anticipatory phase of DA signaling to glial modulation. In the chronic social stress (CSS) model, GRAB-DA fiber photometry recordings demonstrate selective attenuation of NAc DA activity during reward anticipation, while consummatory DA responses remain intact [144]. This anticipatory deficit is tightly correlated with impaired effort allocation in progressive-ratio tasks and delayed reward learning. Given their role in regulating cue encoding, extracellular glutamate/ATP/adenosine balance, and D-serine release, astrocytes are strong candidates for mediating this vulnerability. Failure of astrocytic support during stress likely disrupts terminal excitability and DA release probability, weakening motivational vigor and reward pursuit [144],[145].

### 5.2. Microglial contributions to motivational deficits, anhedonia, and stress

Microglia are also critical in shaping motivational circuits, particularly under chronic stress. Prolonged stress reliably activates microglia across mesocorticolimbic regions, inducing morphological hypertrophy, upregulation of immune-related genes, and remodeling of synaptic architecture in the NAc, PFC, and ventral tegmental area (VTA) [146]. These cellular and molecular changes correlate with impaired reward learning, diminished effort allocation, and increased vulnerability to stress-induced anhedonia. Moreover, psychostimulant exposure under prior stress conditions amplifies microglial reactivity, exacerbating motivational rigidity and addiction-like behaviors [147].

Inflammatory signaling provides a key mechanistic axis for these effects. In chronic unpredictable mild stress (uCMS) paradigms, animals exhibit elevated levels of IL-1β and TNF-α, changes that co-occur with anhedonic behaviors such as reduced sucrose preference [148],[149]. These cytokine elevations are closely linked to dopaminergic pathway disruption, supporting a cytokine-to-DA signaling axis through which neuroinflammation undermines motivation. Clinical studies of major depressive disorder (MDD) reinforce these findings, consistently reporting microglial overactivation and elevated inflammatory markers in affected individuals [150],[151]. Collectively, these insights converge on the view that microglia represent a tractable therapeutic target for restoring motivational drive by modulating dopaminergic signaling and network excitability.

Developmental perspectives further underscore the role of microglial priming in long-term motivational vulnerability. Early-life stress (ELS) is consistently associated with persistent elevations in pro-inflammatory cytokines, dysregulated hypothalamic-pituitary-adrenal (HPA) axis function, and blunted anticipatory reward responses, with findings supported by human neuroimaging and rodent models [152],[153]. These data suggest that microglia undergo priming during sensitive developmental windows, lowering their threshold for inflammatory reactivity to later stressors. Complementary developmental studies demonstrate that microglia regulate NAc synaptogenesis during adolescence, sculpting excitatory-inhibitory balance in motivational circuits. Perturbations of this process through stress, immune activation, or genetic risk can durably reweight mesolimbic connectivity, biasing individuals toward long-term motivational deficits [154]. Together, these findings underscore the developmental origins of motivational pathology, with microglial priming serving as a key mechanistic link between early adversity and later impairments in reward processing.

### 5.3. Glial mechanisms modulating dopamine-dependent motivational circuits

Astrocytic gliotransmission and uptake exert a central influence on motivational encoding. Activity-defined astrocytic ensembles within the NAc, recruited during cue-reward associations, release gliotransmitters such as glutamate, ATP, and D-serine while controlling their clearance from the extracellular space. These processes tune medium spiny neuron excitability and modulate DA terminal release probability, with particularly strong effects during anticipatory epochs of reward processing, thereby shaping motivational drive [113],[141].

Within the GPe, astrocytes regulate computations of the indirect pathway that determines strategy selection. Chemogenetic activation experiments demonstrate that astrocytic signaling in this region suppresses habitual action patterns and biases behavior toward flexible, outcome-sensitive strategies. This astrocytic influence represents a mechanism for balancing motivational persistence with adaptive flexibility under shifting reward contingencies [142].

Microglia likewise shape motivational states through cytokine-DA coupling. Reactive microglia release pro-inflammatory mediators such as IL-1β and TNF-α, which suppress DA synthesis in midbrain neurons, impair receptor expression in the NAc and PFC, and prune dendritic spines. These actions collectively blunt reward anticipation and promote anhedonia. Moreover, framing cytokines as neuromodulators of motivational valence highlights their dual role as immune effectors and circuit-level regulators [155],[156].

Developmental factors further determine the sensitivity of glial-DA interactions. Early-life stress and immune perturbations induce lasting microglial priming, biasing microglial states toward hyper-reactivity upon later stress exposure. This developmental priming alters the maturational trajectory of NAc connectivity and mesolimbic DA signaling, producing enduring phenotypes of reduced effort expenditure and diminished motivational resilience [12].

### 5.4. Behavioral readouts and experimental toolchains for dopamine-glia interactions

Advances in behavioral neuroscience and recording technologies have provided precise readouts of dopamine-glia interactions across motivational and stress paradigms. One of the most transformative has been phase-specific DA monitoring using genetically encoded GRAB-DA sensors in combination with fiber photometry. This approach enables discrimination between anticipatory and consummatory DA signals, revealing that chronic stress selectively attenuates anticipatory activity while sparing consummatory responses, a dissociation that identifies a mechanistic window for glial intervention [49],[51].

Operant behavioral assays complement neurochemical recordings by indexing motivational strategy with high resolution. Furthermore, progressive ratio schedules quantify effort-based responding, while outcome devaluation paradigms distinguish habitual from goal-directed action control [157]. These measures are highly sensitive to astrocytic manipulations within the GPe, where chemogenetic activation shifts behavior away rigid habit formation toward flexible, outcome-driven strategies [142].

Causal approaches extend this framework by directly manipulating astrocytic ensembles. Optogenetic and chemogenetic interventions in activity-defined astrocytic populations of the NAc demonstrate that selective reactivation of these ensembles is sufficient to bias cue-driven approach behavior. Such findings establish that astrocytic ensembles encode motivationally salient information and can directly influence behavioral output [141],[158].

Stress paradigms, including CSS, uCMS, and ELS, provide translationally relevant models for probing the intersection of immune activation with dopaminergic signaling. When combined with cytokine profiling and phase-resolved DA photometry, these paradigms map immune-dopamine-behavior triads, clarifying how systemic inflammation, glial reactivity, and motivational impairments converge [159],[160].

Taken together, this expanding methodological toolchain integrates behavioral assays, real-time neurochemical monitoring, and causal manipulations to generate mechanistically precise and clinically relevant insights into glial regulation of motivation.

### 5.5. Glial-DA crosstalk across disorders: Shared mechanisms of motivation, stress, and neuroinflammation

**Figure 2.**
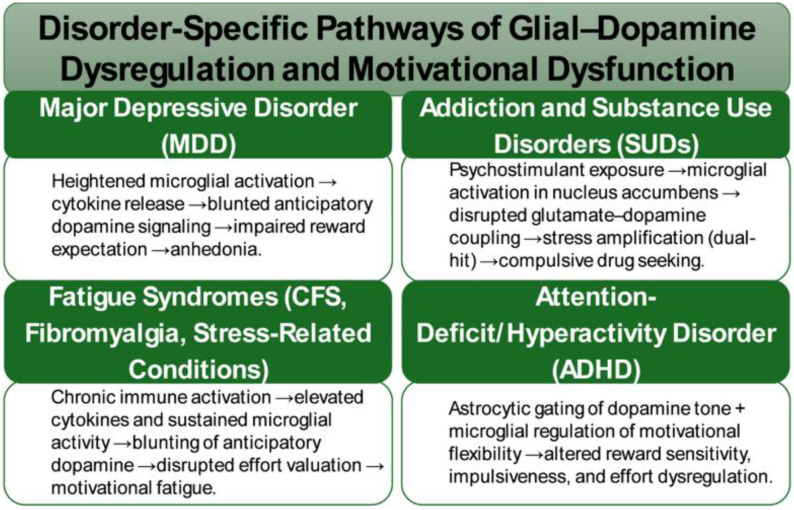
Disorder-specific pathways of Glial-DA dysregulation and motivational dysfunction.

Across psychiatric and stress-related disorders, convergent evidence implicates glial-DA interfaces as central mediators of motivational pathology. In MDDs, human neuroimaging and preclinical models consistently demonstrate attenuated anticipatory DA responses during reward expectation tasks. These deficits are paralleled by heightened microglial activation and amplified cytokine signaling, suggesting that neuroinflammatory cascades act as critical drivers of anhedonia. Additionally, translational studies indicate that interventions targeting glial pathways, including cytokine inhibitors and adenosine A2A receptor modulators, may restore anticipatory DA signaling and thereby alleviate motivational impairments (Figure 2) [25],[161],[162].

In addiction and substance use disorders, psychostimulant exposure robustly engages microglial programs within the nucleus accumbens, disrupting the coupling between glutamate and DA, leading to maladaptive changes in synaptic plasticity and motivational salience. Under conditions of chronic stress, these drug-induced adaptations are exacerbated, creating a dual-hit scenario in which stress-driven microglial activation converges with drug-induced neuroplasticity to reinforce compulsive drug-seeking behaviors. This interplay underscores immune-glial signaling as a central mechanism regulating addiction vulnerability at the nexus of stress reactivity and reinforcement learning [163].

Immune-mediated suppression of anticipatory DA signaling has also been observed in conditions characterized by pathological fatigue and effort intolerance, including chronic fatigue syndrome, fibromyalgia, and stress-related medical syndromes. Elevated cytokines and sustained microglial activation in these contexts appear to blunt reward anticipation and disrupt effort valuation. This integrative framework provides a unifying account of motivational fatigue that bridges psychiatric symptomatology with somatic illness [164],[165].

Emerging insights further suggest a role for glial modulation in attention-deficit/hyperactivity disorder, though causal evidence remains limited. Astrocytic gating of anticipatory DA signaling and microglial regulation of motivational flexibility are hypothesized to contribute to deficits in sustained effort, altered reward sensitivity, and impulsiveness. While speculative, these hypotheses position glial biology as a novel frontier in the mechanistic understanding of ADHD [50],[166].

Despite these advances, fundamental questions remain regarding the precise dynamics by which glial-DA interfaces regulate motivation across stress and disease states. Temporal specificity constitutes a major unresolved dimension: It is unclear which phases of stress exposure, acute versus chronic or anticipatory versus consummatory, are most susceptible to glial modulation. Closed-loop paradigms that integrate real-time DA monitoring with selective glial stimulation hold promise for resolving these dynamics with unprecedented precision. Another critical challenge involves subtype and state specificity. Single-cell and spatial multi-omics have revealed striking heterogeneity between nucleus accumbens shell and core astrocytes, as well as diverse microglial states ranging from homeostatic to pro-inflammatory. Yet, causal links between these transcriptional programs and motivational behaviors remain to be established.

Equally unresolved are the mechanisms by which gliotransmitters contribute to anticipatory gain control. The relative influence of adenosine, glutamate, and D-serine remains poorly defined, and advances will likely require transmitter-specific biosensors combined with astrocyte-restricted manipulations. From a translational standpoint, immune-DA bridges represent especially promising points of intervention. Strategies aimed at modulating IL-1β and TNF-α signaling or tuning adenosine A1/A2A pathways may restore anticipatory DA function and motivational vigor in stress-linked anhedonia, though rigorous clinical testing is lacking.

Finally, developmental factors must be considered, as early-life stress has been shown to prime microglial reactivity and disrupt the maturation of mesocorticolimbic circuitry, establishing long-term vulnerability to motivational pathology. Thus, longitudinal studies integrating immune profiling, multimodal imaging, and behavioral phenotyping will be essential to identify critical preventive windows and to design strategies that recalibrate glial DA coupling before maladaptive trajectories become entrenched.

## 6. Dopamine-Glia crosstalk in neurodegeneration and disease

Pathological states such as neurodegeneration, chronic infection, inflammation, psychological stress, and aging can progressively transform astrocytes and microglia from supportive partners into maladaptive phenotypes [167]. In the dopaminergic system, these shifts converge on mechanisms, including redox imbalance, glutamate dysregulation, mitochondrial and ferroptotic vulnerability, extracellular matrix (ECM) remodeling, and the exosomal propagation of inflammatory signals, eroding neuronal resilience (Table 5) [168],[169].

**Figure 3.**
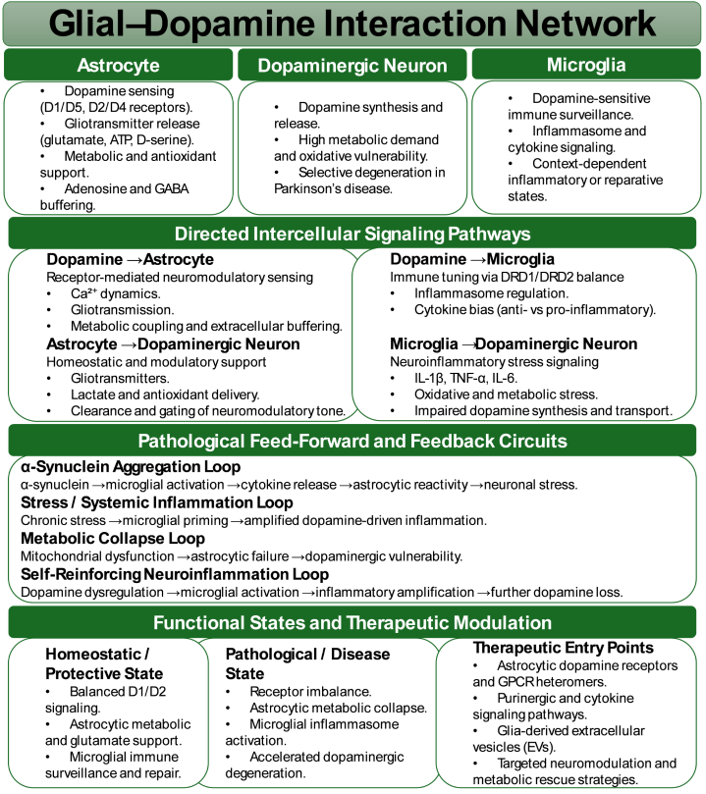
Integrated glial-dopamine interaction network in health and disease.

These convergent mechanisms are summarized in an integrated schematic, illustrating the reciprocal astrocyte-microglia-dopaminergic neuron network and its feed-forward inflammatory and metabolic loops (Figure 3). The advent of high-resolution multi-omics, in vivo imaging, and functional dissection has begun to delineate how these pathways collectively drive dopaminergic circuit fragility [170],[171].

**Table 5.** Integrated mechanisms of dopamine-glia crosstalk in neurodegeneration and disease.

Pathological Context	Core Mechanisms	Key Evidence	Clinical/Pathological Relevance
PD: astrocytic and microglial reprogramming	Astrocytic A1-like conversion (loss of glutamate clearance, trophic support, ↑ oxidative/inflammatory signaling) induced by microglial cytokines (IL-1α, TNF-α, C1q).	Postmortem SN and preclinical models.	Microglia drive astrocytic maladaptation, weakening DA neuron resilience.
Microglial transitions, ECM, and exosomes	CSF1R inhibition (PLX5622) reduces microglia, protects DA neurons, remodels ECM; microglial exosomes carry inflammatory/α-syn cargo, induce A1 astrocytes.	α-syn models; CSF1R and Peli1 manipulations.	Microglia-ECM and vesicular signaling amplify synucleinopathy and degeneration.
Ferroptotic stress	DA neurons are highly vulnerable due to iron turnover, autoxidation, and mitochondrial load. Maladaptive glia exacerbates glutamate dysregulation, redox imbalance, lipid peroxidation.	Ceftriaxone restores SLC7A11/GPX4; multi-omics link glia to ferroptosis.	Identifies ferroptosis as glia-amplified death pathway; therapeutic targets include GPX4 activators and cystine-glutamate modulators.
Post-viral (Long COVID) basal ganglia dysfunction	Chronic inflammation disrupts corticostriatal DA loops: ↓ synthesis/release, ↓ receptor sensitivity; BBB disruption, astrocytic endfoot injury sustain glial priming.	Neuroimaging of basal ganglia; ↑ GFAP, IL-6, TNF-α in post-COVID cohorts.	Explains fatigue, effort intolerance, cognitive slowing via DA insufficiency and glial-driven basal ganglia failure.
Aging as an amplifier	Aged microglia adopt primed pro-inflammatory states; astrocytes show impaired glutamate clearance, reduced lactate/antioxidant support, ↑ ROS/lipid peroxidation.	Microglial/astrocytic aging phenotypes; PD datasets show stronger inflammatory signatures in older cohorts.	Aging synergizes with inflammation and metabolic stress, accelerating dopaminergic degeneration.

### 6.1. Glial-DA interfaces in PD: Inflammatory reprogramming and ferroptotic stress

In PD, astrocytes undergo marked state transitions that critically influence dopaminergic neuron survival. Postmortem analyses and preclinical models consistently demonstrate the accumulation of A1-like, or “neurotoxic”, astrocytic programs within substantia nigra microenvironments [172]–[174]. These astrocytes exhibit heightened inflammatory and oxidative profiles, impaired EAAT2/GLT-1-mediated glutamate clearance, and diminished trophic and metabolic support [175]. Scientometric analyses of the PD-astrocyte literature highlight the rapid rise of A1 conversion as a conceptual anchor, reflecting growing recognition that astrocytic phenotype switching is mechanistically linked to dopaminergic vulnerability [176].

Microglia are principal drivers of this maladaptive transition. Cytokines such as IL-1α, TNF-α, and C1q induce A1 polarization, causing astrocytes to lose essential homeostatic functions, including glutamate buffering, potassium regulation, and metabolic shuttling, while gaining complement- and cytokine-mediated neurotoxicity [177]. These changes are especially damaging in the substantia nigra, where the oxidative burden of DA metabolism imposes intrinsic stress. Importantly, activated microglia is necessary for A1 induction, positioning microglial signaling upstream of astrocytic maladaptation [177],[178].

Microglial state transitions are central to PD progression. In α-synuclein overexpression models, inhibition of the colony-stimulating factor 1 receptor (CSF1R) with PLX5622 reduces microglial numbers, attenuates dopaminergic neurodegeneration, improves motor outcomes, and remodels ECM-associated transcriptional networks [179],[180]. These findings implicate microglia-ECM crosstalk as a determinant of synaptic and circuit integrity [181]. In addition, microglia-derived exosomes function as carriers of inflammatory and pathological cargo, including molecules that convert astrocytes into A1-like states. Disrupting this vesicular axis through targets such as Peli1 has been proposed as a strategy to interrupt glia-to-glia amplification loops [182],[183].

Converging evidence from seeded α-synuclein models further indicates that microglial depletion via CSF1R blockage attenuates α-syn propagation and protects dopaminergic neurons, reinforcing the concept that microglia act as gatekeepers of synucleinopathy spread [180].

The intrinsic bioenergetic and metabolic architecture of dopaminergic neurons renders them acutely susceptible to ferroptotic death. Elevated iron turnover, catecholamine autoxidation, and the exceptionally high oxidative load of mitochondrial respiration collectively create a ferroptosis-permissive milieu [184]. Within this context, maladaptive astrocytic and microglial states act as critical amplifiers of ferroptotic stress. Experimental pharmacology underscores this vulnerability: Treatment with ceftriaxone attenuates glial activation while suppressing ferroptosis by restoring SLC7A11/GPX4 antioxidant defenses [185]. These effects translate into robust preservation of dopaminergic viability in both in vitro and in vivo models of PD, thereby linking astrocytic glutamate clearance, redox homeostasis, and iron-lipid peroxidation dynamics to actionable therapeutic targets [186].

Moreover, multi-omics and network-level studies have further mapped iron homeostasis and lipid peroxidation cascades onto astrocytic and microglial gene regulatory programs, identifying glial populations as arbiters of ferroptotic thresholds in dopaminergic circuits [187],[188]. This emerging framework provides a compelling rationale for glia-directed anti-ferroptotic interventions. Strategies under investigation include GPX4 activators, modulators of cystine-glutamate exchange, and approaches aimed at stabilizing astrocytic antioxidant and metabolic defenses. Together, these insights delineate a promising therapeutic frontier in the modification of PD progression.

### 6.2. Immune-Glial coupling and DA circuit failure: Basal ganglia pathophysiology in post-COVID states

Accumulating evidence indicates that viral infections, particularly SARS-CoV-2, exert lasting impacts on basal ganglia circuits, with significant consequences for dopaminergic signaling [189]. Multimodal neuroimaging studies consistently identify structural and functional alterations in the putamen, pallidum, and caudate nucleus, including reduced gray matter volume, disrupted metabolic activity, and impaired functional connectivity [190]. These abnormalities correlate with clinical phenotypes of Long COVID (fatigue, effort intolerance, motivational deficits, and cognitive slowing) symptoms that map directly onto dopamine-dependent computations of reward valuation and cognitive vigor.

Mechanistically, these outcomes converge on disruption of corticostriatal loops central to effort-reward integration. Sustained neuroinflammation, driven by persistent immune activation, is proposed to impair dopaminergic function through multiple suppression of DA synthesis, reduction in release probability, and downregulation of receptor sensitivity [191],[192]. Such immune-driven imbalances establish a state of dopaminergic insufficiency under chronic inflammatory pressure.

Biomarker studies provide further support for this framework, consistently reporting elevated glial fibrillary acidic protein (GFAP), indicative of astrocytic injury, alongside pro-inflammatory cytokines, including IL-6 and TNF-α, in post-COVID cohorts [193],[194]. Evidence of blood-brain barrier (BBB) disruption, mediated by inflammatory endothelial signaling and astrocytic endfoot dysfunction, reinforces the view of systemic-central immune coupling as a key driver of dopaminergic vulnerability. In this model, peripheral cytokine tone sustains central glial priming, which alters basal ganglia homeostasis through tetrahydrobiopterin depletion, dysregulated dopamine transporter (DAT) kinetics, and reduced D1/D2 receptor availability. The outcome is a failure of neuromodulatory gain control within corticostriatal circuits, providing a mechanistic substrate for the motivational and cognitive deficits that characterize Long COVID [131],[194].

### 6.3. Aging and the Glial-DA interface: Dynamic amplification of neuroinflammatory and metabolic stress

Aging functions not merely as a background risk factor but as a biological amplifier of glial-DA dysfunction. Microglia in aged brains adopt a “primed” phenotype, marked by lower activation thresholds and a pro-inflammatory bias. This state leads to exaggerated responses to secondary insults, such as viral infections or α-synuclein aggregates, and impairs resolution of inflammation, thereby prolonging dopaminergic stress exposure [73],[195].

Astrocytic decline with age compounds this vulnerability. Aging astrocytes display impaired glutamate clearance, reduced lactate shuttling, diminished antioxidant buffering, and altered calcium signaling [127]. These deficits undermine metabolic and synaptic support for DA neurons, promoting excitotoxic and oxidative stress. Concurrently, age-related mitochondrial decline in glial populations enhances reactive oxygen species (ROS) accumulation and lipid peroxidation, amplifying damage to dopamine-rich circuits [196].

Scientometric analyses of PD datasets emphasize the age-reactivity interaction, demonstrating that older cohorts exhibit more pronounced inflammatory astrocytic signatures and accelerated dopaminergic degeneration [197]. Together, these findings establish aging as a dynamic amplifier of maladaptive glial states, converging with neuroinflammation, mitochondrial stress, and systemic insults to erode dopaminergic resilience.

### 6.4. Glial reprogramming as a convergent amplifier of dopaminergic dysfunction

Across PD, viral insults, such as Long COVID, and aging show that several mechanistic axes converge to illustrate how glial reprogramming amplifies dopaminergic dysfunction.

A central pathway is the microglia-to-astrocyte signaling cascade, in which activated microglia secrete IL-1α, TNF-α, and C1q, driving the conversion of astrocytes into A1 neurotoxic states. Once reprogrammed, these astrocytes lose essential homeostatic functions, including glutamate buffering, potassium regulation, and trophic/metabolic support while acquiring neurotoxic complement and cytokine activities. This switch establishes a feed-forward inflammatory loop that accelerates dopaminergic degeneration, particularly in oxidative-stress-vulnerable regions such as the substantia nigra [177].

Another recurrent theme is ECM remodeling as a disease amplifier. In α-synuclein models, pharmacological depletion of microglia through CSF1R inhibition not only attenuate dopaminergic neurodegeneration but also reprogram ECM networks. These findings highlight that ECM-microglia crosstalk is a decisive determinant of synaptic stability and axonal survival, positioning ECM remodeling as a potential therapeutic axis to stabilize nigrostriatal architectures [198],[199].

Glial communication is also propagated through exosomal transfer of pathological cargo. Microglia release (EVs) enriched in pro-inflammatory proteins, miRNAs, and α-syn aggregates induces astrocytic conversion toward A1-like states and propagates neuroinflammatory tone across local microenvironments. Targeting pathways of exosome biogenesis, trafficking, and uptake therefore offers a tractable opportunity to disrupt this glia-glia amplification loop and mitigate progressive dopaminergic vulnerability [200],[201].

A further point of convergence lies in ferroptosis coupling to glial metabolism. Dopaminergic neurons are intrinsically ferroptosis-prone due to their high oxidative load, iron turnover, and catecholamine autoxidation. Glia modulates this vulnerability through SLC7A11/GPX4 antioxidant pathways and astrocytic GLT-1-mediated glutamate clearance, both of which serve as critical buffers against iron-lipid peroxidation. Pharmacological interventions such as ceftriaxone provide the proof-of-concept that enhancing these pathways suppresses lipid peroxidation and ferroptotic stress, thereby conferring neuroprotection in dopaminergic circuits [202]–[205].

Finally, immune priming and BBB dysfunction emerge as unifying features in chronic inflammatory states. Biomarker studies in Long COVID consistently report elevated GFAP, increased systemic cytokine tone, and markers of BBB leak, collectively reflecting prolonged glial activation and impaired dopaminergic resilience [206],[207]. BBB disruption thus represents a mechanistic driver of central vulnerability and a measurable endpoint for risk stratification and therapeutic monitoring.

Taken together, these convergent mechanisms position glia as active amplifiers of dopaminergic stress across neurodegeneration, viral sequelae, and aging. By integrating cytokine signaling, ECM remodeling, exosomal transfer, ferroptotic coupling, and barrier dysfunction, researchers reframe dopaminergic vulnerability as a systems-level phenomenon in which glial states orchestrate the trajectory from resilience to degeneration.

### 6.5. Reframing glial therapeutics: Targeting microglial and astrocytic programs in PD and beyond

**Figure 4.**
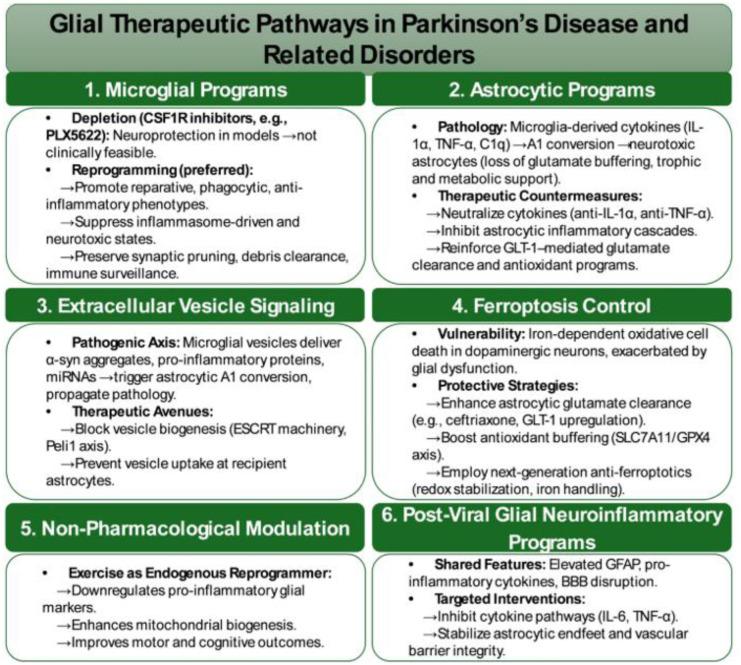
Emerging glial therapeutic pathways: Precision modulation of microglial and astrocytic programs in PD and related disorders.

Therapeutic efforts targeting dopamine-glia interactions are moving beyond broad immunosuppression toward approaches that emphasize state reprogramming, pathway modulation, and resilience promotion (Figure 4). Preclinical depletion of microglia using CSF1R inhibitors such as PLX5622 has demonstrated robust neuroprotection and attenuation of α-synuclein pathology [208]. However, complete ablation is neither feasible nor desirable in humans, as microglia are indispensable for immune surveillance, synaptic remodeling, and debris clearance. Consequently, translational strategies prioritize microglial reprogramming, seeking to bias these cells toward reparative, phagocytic, and anti-inflammatory phenotypes while suppressing inflammasome-driven and neurotoxic states [73],[115].

Parallel efforts focus on preventing astrocytic conversion into maladaptive A1-like states, a consistent feature across Parkinsonian models. This transition, driven by microglia-derived cytokines, such as IL-1α, TNF-α, and C1q, deprives astrocytes of critical homeostatic functions and confers neurotoxic activity. Therapeutic avenues under development include cytokine neutralization, selective inhibition of astrocytic inflammatory pathways, and reinforcement of metabolic and trophic programs that preserve homeostatic astrocyte functions [209].

Interventions at the level of extracellular vesicle signaling are also gaining prominence. Microglia-derived vesicles transfer α-syn aggregates, pro-inflammatory proteins, and miRNAs that trigger astrocytic A1 conversion and propagate neuroinflammation across dopaminergic territories [183]. Strategies aimed at disrupting vesicle biogenesis, such as targeting ESCRT machinery or the Peli1 axis, or blocking vesicle uptake at recipient astrocytes, represent novel ways to interrupt glia-to-glia amplification loops and contain the spread of pathology.

A further therapeutic channel involves controlling ferroptosis, an iron-dependent form of cell death exacerbated in dopaminergic neurons by high oxidative stress and catecholamine autoxidation. Glial regulation of glutamate homeostasis through GLT-1 and antioxidant buffering via the SLC7A11/GPX4 pathway provides critical protection against this process. Ceftriaxone enhances astrocytic glutamate clearance and antioxidant activity, thereby reducing excitotoxicity and ferroptosis [205],[210]. Beyond this, next-generation anti-ferroptotic compounds designed to stabilize glial redox capacity and iron handling are advancing as promising translational candidates [188].

Non-pharmacological interventions such as exercise add an important dimension, functioning as endogenous modulators of glial states. Aerobic and resistance training in experimental Parkinson's models have been shown to downregulate pro-inflammatory astrocytic and microglial markers, enhance mitochondrial biogenesis, and improve motor outcomes [211],[212]. Thus, exercise acts as a physiological reprogrammer, shifting glia toward pro-homeostatic phenotypes and reinforcing dopaminergic resilience.

Post-viral syndromes, including Long-COVID, highlight another translational frontier: Patients consistently exhibit elevated GFAP, pro-inflammatory cytokines, and BBB disruption [207],[213]. Thus, targeting cytokine signaling pathways, such as IL-6 and TNF-α, with strategies to stabilize astrocytic endfeet and endothelial barrier integrity provides a rational approach to mitigating chronic glial priming and dopaminergic fragility under conditions of sustained immune activation.

Despite these promising advances, key challenges remain. A central priority is the identification of predictive biomarkers, including GFAP, neurofilament light chain (NfL), extracellular matrix fragments, and glia-derived vesicular cargo, which can forecast dopaminergic decline prior to irreversible nigrostriatal loss. Equally critical is the delineation of resilience programs, as astrocytic metabolic pathways, such as lactate shuttling and antioxidant buffering, and microglial phenotypes that preserve synaptic integrity without inflammasome activation may sustain long-term dopaminergic stability. Comparative multi-omics across PD and Long-COVID cohorts are also required to determine whether a shared vulnerability transcriptome, spanning cytokine signaling, extracellular matrix remodeling, and vesicular pathways, underlies cross-disorder risk. Finally, therapeutic precision must be optimized, with the timing, dosing, and safety of interventions such as CSF1R modulators, anti-A1 conversion agents, and anti-ferroptotic compounds rigorously defined in clinical settings and supported by biomarker evidence demonstrating on-target glial reprogramming in humans.

Collectively, these emerging strategies redefine the therapeutic horizon for dopamine-glia biology. The field is shifting from indiscriminate suppression toward precision reprogramming, with the objective of stabilizing glial ecosystems that support dopaminergic resilience across stress, disease, and aging.

## 7. Glia-directed therapeutic paradigms in dopaminergic neurodegeneration

**Figure 5.**
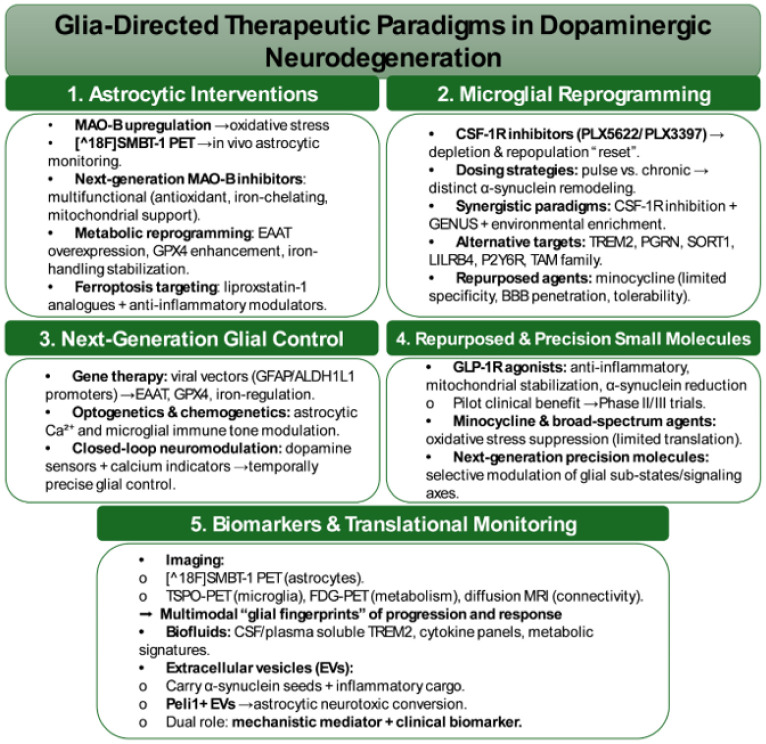
Glia-directed therapeutic paradigms in dopaminergic neurodegeneration.

Evidence increasingly positions glial state transitions, rather than neuronal degeneration alone, as the primary inflection point for dopaminergic circuit vulnerability. This recognition is transforming therapeutic paradigms from neuron-centric rescue toward glia-directed interventions that normalize astrocytic metabolism, restrain maladaptive microglial reactivity, and enable longitudinal monitoring of glial dynamics through molecular imaging and fluid biomarkers (Figure 5) [12],[214]. By reframing therapeutic entry points at the astrocyte-microglia interface, interventions can be positioned upstream of irreversible neuronal loss, thereby extending the window for disease modification.

### 7.1. Astrocytic interventions in Glial-DA interfaces

Astrocytes occupy a central role in this therapeutic shift, given their regulation of synaptic glutamate concentrations via EAAT1/2 and their control of oxidative tone through monoamine oxidase-B (MAO-B) activity. In reactive states, MAO-B expression is markedly upregulated, functioning as a driver of oxidative stress and as a tractable theranostic target [215],[216]. Advances in astrocyte-selective PET imaging underscore this translational opportunity. The tracer [^18F] SMBT-1 exhibits highly selective and reversible binding to MAO-B with minimal nonspecific uptake, enabling longitudinal quantification of reactive astrogliosis [217]. Initial human studies confirmed favorable kinetic properties, and more recent preclinical applications extended their utility to amyloid and tau transgenic models, where tracer signals correlated strongly with histological indices of astrocytic reactivity [218]. Importantly, SMBT-1 is being integrated into multi-tracer paradigms, where astrocytic imaging is combined with TSPO-PET (for microglial activation) and FDG-PET (for metabolic flux), generating multidimensional “glial fingerprints” of disease trajectory and therapeutic response [219].

Therapeutic efforts are proceeding in parallel with next-generation MAO-B inhibitors. Unlike classical agents that act solely on enzymatic suppression, newer compounds incorporate antioxidant scaffolds, iron-chelating groups, and mitochondrial-supportive motifs. These multifunctional designs simultaneously mitigate upstream enzymatic overactivity and downstream cascades of reactive oxygen species and lipid peroxidation [220],[221]. Complementing pharmacologic inhibition, astrocytic metabolic reprogramming has emerged as a broader strategy, targeting mitochondrial dynamics, lipid metabolism, and glutathione-dependent antioxidant defenses. Modalities include viral vectors driving EAAT overexpression, small molecules enhancing GPX4 activity, and interventions that stabilize iron-handling proteins, collectively positioning astrocytes as pivotal hubs of dopaminergic neuroprotection [221].

Ferroptosis has become a particularly salient frontier, defined as an iron-dependent cell death pathway driven by glutathione depletion and phospholipid peroxidation. While dopaminergic neurons are intrinsically ferroptosis-prone due to their metabolic profile, maladaptive astrocytic and microglial states further amplify this vulnerability. Dysregulated iron sequestration and lipid metabolism within glia accelerate oxidative stress and destabilize nigrostriatal circuits [222]. Moreover, studies indicate that microglial cytokine signaling can disrupt astrocytic iron handling, leading to the accumulation of labile iron pools that precipitate peroxidative damage. This establishes a self-reinforcing loop in which iron-driven lipid peroxidation fuels neuroinflammation, further compounding dopaminergic fragility [223],[224].

As a result, therapeutic paradigms are shifting toward combinatorial approaches that jointly target oxidative and inflammatory axes. Ferroptosis inhibitors, such as liproxstatin-1 analogues, are being paired with anti-inflammatory modulators to simultaneously suppress lipid peroxidation and restrain cytokine-driven propagation of neuroinflammation [225]. Such dual targeting is increasingly recognized as essential to disrupting the glia-iron-inflammation cycle that underlies Parkinsonian progression.

### 7.2. Microglial reprogramming: Inhibitors, modulators, and immune tuning

Therapeutic strategies increasingly recognize that targeting microglial states rather than broadly suppressing neuroinflammation offers a more refined approach to preserving dopaminergic resilience. Among the most extensively studied interventions are CSF-1R inhibitors, including PLX5622 and PLX3397 [226]. Sustained administration of these agents in preclinical Parkinsonian models induces near-complete depletion of resident microglia, which attenuates α-synuclein accumulation, dopaminergic neuron loss, and motor dysfunction [179]. Notably, dosing regimens critically shape outcomes: While chronic depletion reduces pathology, shorter pulse exposures reorganize α-synuclein inclusions into fewer but larger aggregates, thereby altering clearance demands and circuit remodeling trajectories [227]. Following drug withdrawal, microglial repopulation arises from central nervous system progenitors, generating a population with reduced inflammatory tone and enhanced trophic support [228]. This “reset” phenotype suggests that temporally controlled depletion-repopulation cycles may function as a reprogramming strategy with long-term therapeutic relevance.

More recent combinatorial approaches have integrated CSF-1R inhibition with neuromodulatory interventions such as gamma entrainment using sensory stimulation (GENUS) and environmental enrichment. These paradigms demonstrate synergistic effects, restoring network synchrony, enhancing learning and memory, and accelerating recovery in preclinical models [229]. Such findings highlight the potential for immune modulation and circuit-level entrainment to act in concert, recalibrating dopaminergic networks through coordinated glial and neuronal plasticity.

Beyond CSF-1R signaling, a growing repertoire of microglial pathways has emerged as therapeutic entry points. Receptors such as TREM2, progranulin (PGRN), SORT1, LILRB4, P2Y6R, and TAM family members regulate phagocytosis, lipid metabolism, apoptotic clearance, and cytokine release [230]. Modulation of these axes can bias microglia toward reparative states that support synaptic integrity and metabolic homeostasis while restraining feed-forward astrocytic conversion into neurotoxic phenotypes. In parallel, pharmacological repurposing efforts have entailed broad-spectrum agents, such as minocycline, which reduce oxidative stress, dampen pro-inflammatory cytokine release, and disrupt maladaptive astrocyte-microglia feedback loops [231]. Yet, despite extensive preclinical efficacy, clinical translation has been limited by concerns regarding specificity, blood-brain barrier penetrance, and sustained efficacy. These limitations underscore the need for next generation immunomodulators designed with state selectivity and precise temporal control.

### 7.3. Next-generation glial control: Gene therapy, optogenetics, and chemogenetics

The convergence of molecular engineering and circuit neuroscience is enabling direct manipulation of glial physiology with unprecedented precision. Advances in viral vector technology, particularly those leveraging astrocyte-selective promoters, such as GFAP and ALDH1L1, enable targeted delivery of therapeutic transgenes to glial populations [232]. This approach has been applied to augment glutamate clearance through EAAT overexpression, bolster antioxidant defenses via GPX4, and stabilize iron homeostasis by enhancing expression of iron-handling proteins, thereby restoring astrocytic buffering capacity against excitotoxic and oxidative stress.

Complementing gene therapy, optogenetic and chemogenetic strategies provide causal control of glial contributions to dopaminergic circuitry. Manipulation of astrocytic Ca²⁺ dynamics can bidirectionally regulate synaptic plasticity, while DREADD-based modulation of microglia reconfigures immune tone and alters dopaminergic responsiveness in vivo [233]. With the integration of genetically encoded DA sensors, calcium indicators, and closed-loop control systems, these tools can map and manipulate glial activity in temporally and spatially defined behavioral epochs [234]. Such approaches position astrocytic and microglial neuromodulation not only as experimental probes of causality but also as potential therapeutic modalities that are capable of precision reprogramming of glial states in circuit-defined contexts relevant to PD and related dopaminergic disorders.

### 7.4. Glial biomarkers and translational monitoring in dopaminergic circuit pathophysiology

Advances in molecular imaging and fluid biomarker development are redefining how glial dynamics in dopaminergic circuits are monitored and translated into clinical frameworks. Among these, astrocytic MAO-B PET tracers, particularly [^18F] SMBT-1, have emerged as highly selective and non-invasive tools for quantifying astrocyte reactivity in vivo, as clinical studies confirm favorable pharmacokinetics, binding specificity, and reversibility, supporting their integration into longitudinal interventional trials [217],[235]. Preclinical investigations further validate SMBT-1 by demonstrating close correspondence between tracer uptake and histological burden in transgenic amyloid and tau models, establishing its face validity as a biomarker of glial engagement [236].

An increasingly powerful paradigm involves multimodal imaging, in which astrocytic MAO-B PET is combined with TSPO-PET for microglial activation and metabolic or connectivity measures such as FDG-PET and diffusion MRI. These integrated pipelines generate multicellular progression signatures that distinguish disease stages, identify vulnerable network nodes, and track therapeutic response [237],[238]. Complementary neuropathological studies mapping GFAP and other astrocytic markers across Parkinsonian brains further anchor these imaging endpoints as validated measures of regional progression [239],[240].

Beyond imaging, biofluid assays represent an essential translational layer. Cerebrospinal fluid (CSF) and plasma studies increasingly support soluble TREM2, inflammatory cytokine panels, and metabolic microglial signatures as reproducible indicators of disease activity and treatment response [241],[242]. Thus, standardization of biomarker panels and longitudinal sampling protocols is critical to ensure reproducibility across cohorts and facilitate trial stratification.

Additionally, EVs have gained particular attention for their dual role as mechanistic mediators and biomarkers. Microglia-derived EVs carry α-synuclein seeds and pro-inflammatory cargo, directly inducing astrocytic conversion into neurotoxic phenotypes and amplifying dopaminergic stress. Moreover, findings reveal Peli1-containing EV cargo as a driver of astrocytic reactivity, underscoring exosomal pathways as actionable therapeutic targets and minimally invasive biomarkers for clinical monitoring [243],[244].

### 7.5. Repurposed therapeutics and precision small-molecule modulators of Glial-DA pathophysiology

The therapeutic landscape increasingly recognizes repurposed agents as glia-active neuromodulators capable of buffering dopaminergic vulnerability. Glucagon-like peptide-1 receptor agonists (GLP-1RAs), developed originally for diabetes, have shown striking promise in preclinical and early clinical studies. In experimental systems, GLP-1RAs suppress microglial cytokine release, reprogramming microglia toward homeostatic phenotypes while enhancing astrocytic metabolic support by stabilizing mitochondrial function, augmenting glucose uptake, and mitigating excitotoxic stress [245]–[247]. Furthermore, clinical pilot data in PD suggest improvements in motor and cognitive outcomes, spurring multiple Phase II/III trials aimed at clarifying efficacy and mechanistic underpinnings [248]. Mechanistically, GLP-1RAs reduce α-synuclein aggregation and suppress downstream pro-inflammatory cascades, reinforcing their position within a glial-centric therapeutic framework [249].

Other agents, including minocycline, remain within the therapeutic repertoire due to their capacity to attenuate oxidative stress, inhibit microglial pro-inflammatory cascades, and disrupt astrocyte-microglia amplification loops [250],[251]. However, their translation has been constrained by limited specificity, modest long-term tolerability, and concerns over systemic immune suppression. These limitations highlight the urgency of advancing precision small molecules capable of targeting discrete glial sub-states or signaling axes.

Despite growing therapeutic momentum, several translational challenges persist. Timing is critical; microglial modulation through CSF-1R targeting and astrocytic buffering of metabolic stress are most effective in prodromal or early disease phases, necessitating pre-symptomatic detection via PET imaging (MAO-B, TSPO) and fluid biomarkers (EV cargo, soluble TREM2, cytokine panels). Additionally, selectivity is another hurdle, as broad microglial depletion suppresses pathogenic reactivity but risks compromising essential homeostatic functions such as synaptic refinement and debris clearance. Consequently, state-specific interventions and optimized dosing regimens are being prioritized to preserve beneficial roles while curbing maladaptive phenotypes.

Furthermore, heterogeneity of glial states further complicates therapeutic design. Single-cell and spatial transcriptomics reveal striking diversity between astrocytic and microglial sub-states across brain regions, disease stages, and demographic backgrounds, underscoring the need for cell-resolved targeting strategies guided by molecular classifiers and regional imaging markers. Finally, biomarker validation remains a pressing bottleneck. Candidate markers, including MAO-B PET and exosomal cargo signatures, such as Peli1, soluble TREM2, and cfDNA fragments, require rigorous cross-platform validation, longitudinal reproducibility testing, and confirmation in ancestrally and clinically diverse populations. Without such harmonization, translation risks stagnation despite strong preclinical foundations.

## 8. Future directions: Emerging priorities in Glial-DA biology

Glial-DA interactions are recognized as systems-level regulators of behavior, plasticity, and disease vulnerability, though critical gaps remain. Regional heterogeneity, demographic moderators, computational formalization, and translational applications represent key frontiers. Systematically addressing these dimensions will establish a mechanistic foundation for targeted interventions in psychiatric and neurodegenerative disorders.

### 8.1. Regional specificity: Striatum, PFC, hippocampus, and beyond

Large-scale transcriptomic deconvolution of healthy human brain tissue reveals striking region- and sex-specific differences in glial composition. Analyses of GTEx datasets (>1600 samples) indicate that astrocytic fractions increase with age in males across select cortical regions, whereas microglial proportions rise in females, suggesting baseline gradients that shape DA-glia coupling. These patterns are particularly relevant to cortico-striatal and hippocampal loops, where DA dynamics are tuned by astrocytic buffering and microglial surveillance.

The disproportionate vulnerability of SNc neurons in PD relative to VTA neurons is increasingly attributed to regional differences in astrocytic metabolic support, microglial inflammatory thresholds, and vascular-oxidative load. However, causal human evidence remains limited. Similarly, DA-glia interactions in the PFC and hippocampus remain underexplored, despite their established roles in executive control, working memory, and contextual learning.

In future studies, researchers should combine region-resolved single-cell and spatial multi-omics with in vivo DA measurements to define glial receptor repertoires, metabolic programs, and inflammatory thresholds across nuclei. Behavioral paradigms selectively probing PFC (working memory), striatum (reward learning), and hippocampus (contextual encoding) could yield region-specific signatures, closing a major translational gap.

### 8.2. Sex, age, and ancestry effects in Glial-DA coupling

Sex differences in glial biology are robust and multifaceted. Syntheses highlight sexually dimorphic microglial morphology and functional states across cortex, basal ganglia, and hippocampus, largely modulated by hormonal context. Astrocytic density and morphology also show sex-linked variation, with implications for synaptic support and neuromodulation. These cellular distinctions map onto DA dynamics: Fast-scan cyclic voltammetry demonstrates greater DA release in the nucleus accumbens core of females, alongside differential regulation in the dorsolateral striatum, underscoring sex-dependent tuning of reward circuits.

Aging further reshapes glial-DA interactions. Declines in astrocytic glutamate clearance and metabolic support, coupled with microglial priming toward pro-inflammatory states, progressively erode DA signal fidelity. However, longitudinal human datasets linking glial aging trajectories to DA function remain absent, marking an urgent research priority.

In contrast, ancestry-linked variability in glial-DA coupling is virtually unexplored. While population-level variation influences PD prevalence, drug response, and genetic architecture, no researchers directly address ancestry-driven differences in astrocytic receptor expression, microglial activation thresholds, or DA-glia integration. Precision neuroscience must therefore prioritize integrative approaches that account for sex, age, and ancestry to improve generalizability and therapeutic equity.

### 8.3. Computational and AI-driven models

Computational neuroscience has recently begun incorporating glial dynamics into normative frameworks. The 3M-Progress model, an embodied reinforcement learning paradigm, integrated neural-glial modules to reproduce neural and astrocytic signatures underlying futility-induced passivity in zebrafish, representing the first whole-brain normative model of behavior grounded in neural-glial coupling.

In contrast, most neuron-astrocyte models remain limited to Ca²⁺ dynamics, gliotransmitter buffering, or homeostatic regulation, with minimal consideration of reactive state transitions or DA-specific modulation. In future frameworks, researchers should explicitly incorporate glial state variables; astrocytic Ca²⁺ flux, extracellular metabolite concentrations, and microglial inflammatory activity into reinforcement learning architectures using eligibility traces and distributional value functions. Such integration would capture the slow, spatially diffuse modulatory effects of glia that shape DA thresholds, plastic windows, and motivational drive under stress or immune challenges.

Moreover, advances in large-scale neuron-astrocyte simulation platforms demonstrate the feasibility of embedding glia into whole-brain models. These tools could enable predictive simulations of DA decline across aging, PD, and stress-linked pathology, bridging mechanistic insights with clinical applications.

### 8.4. Translational roadmap: From bench to clinic

The translation of glial-DA research into clinical practice requires a multipronged strategy spanning imaging technologies, peripheral biomarker discovery, pharmacological innovation, and behavioral assay development. Progress has been most notable in the imaging domain, where astrocyte-selective PET tracers, such as [^18F] SMBT-1, targeting MAO-B have demonstrated favorable kinetics and reproducibility in PD and Alzheimer's disease cohorts. These tools represent a significant advancement in the ability to non-invasively monitor astrocytic states in vivo. For microglial imaging, TSPO PET remains the most widely used approach; however, its interpretability is constrained by the rs6971 polymorphism. Despite this limitation, post-mortem validation studies have confirmed TSPO binding in progressive supranuclear palsy and Alzheimer's disease, supporting its conditional clinical utility. The development of next-generation ligands targeting receptors such as P2X7 offers the promise of more specific inflammatory readouts, though large-scale clinical validation is required.

Parallel to imaging efforts, the search for peripheral biomarkers of glial-DA coupling is accelerating. Brain-derived EVs enriched in glial proteins and microRNAs, isolated from plasma, have shown diagnostic potential in PD, offering a minimally invasive window into central nervous system dynamics. Complementary approaches using cell-free DNA methylation signatures provide an additional means of inferring neural cell-type origins, enabling real-time monitoring of astrocytic and microglial turnover. The integration of peripheral assays with multimodal imaging platforms, such as PET and MRI, anchored to single-cell and spatial transcriptomic datasets, represents a critical next step. Such convergence will enable the precise mapping of glial states to dopaminergic vulnerability niches across the substantia nigra, ventral tegmental area, and striatal subdivisions, thereby refining diagnostic accuracy and disease staging.

Pharmacological interventions targeting glial pathways in dopamine-related disorders are also progressing, though challenges remain. GLP-1 receptor agonists have emerged as promising agents with central anti-inflammatory actions and preliminary evidence of motor and cognitive benefits in PD. Yet, heterogeneity in clinical outcomes underscores the need for biomarker-guided evaluation to identify responsive subgroups. Conversely, broad-spectrum agents such as minocycline reliably attenuate microglial activation in preclinical models but have failed to deliver consistent disease-modifying benefits in clinical trials, emphasizing the importance of developing more selective compounds and robust markers of target engagement. Future trials will need to incorporate multimodal biomarker panels to ensure mechanistic validation and to facilitate precision stratification of patients.

Beyond pharmacology, behavioral paradigms are increasingly recognized as valuable translational tools for probing glial-DA interactions. Tasks indexing reward anticipation, effort-based decision-making, and fatigue provide ecologically relevant readouts that can be directly linked to underlying astrocytic and microglial modulation of dopaminergic tone. When integrated with glia-selective imaging or peripheral biomarker assays, these paradigms may serve as surrogate endpoints in clinical trials, enabling rigorous assessment of therapeutic effects on glial-DA coupling. Such approaches not only bridge mechanistic discovery with clinical application but also hold the potential to accelerate the development of targeted interventions aimed at restoring circuit-level function and improving patient outcomes.

## 9. Conclusions

In recent years, converging evidence from cellular, genetic, imaging, and systems-level studies has repositioned glial-DA crosstalk as a central regulator of behavior, plasticity, and disease, rather than a secondary adjunct to neuronal signaling. Astrocytes and microglia actively sense, gate, and sculpt dopaminergic circuits through receptor signaling, metabolic support, gliotransmission, and immunometabolic coupling. Mechanistic insights, such as astrocytic MAO-B expression visualized via [^18F] SMBT-1 PET imaging, microglial manipulation with CSF-1R inhibitors, and glial regulation of ferroptosis, demonstrate that glia directly calibrate dopaminergic tone, neuronal vulnerability, and resilience. These findings collectively redefine DA, not as a purely neuronal signal, but as a glia-conditioned output, shaped by local inflammatory states, metabolic buffering, and extracellular matrix dynamics.

This reframing underscores that the efficacy of DA signaling is not fixed but dynamically shaped by glial modulation. The selective vulnerability of dopaminergic populations, such as substantia nigra versus ventral tegmental area neurons, reflects regionally distinct glial phenotypes that determine differential resilience or susceptibility. Likewise, behavioral outcomes, including motivation, anhedonia, and stress resilience, are increasingly recognized as contingent on the functional state of glia rather than neuronal activity alone. Consequently, therapeutic strategies that focus exclusively on dopaminergic neurons are unlikely to be sufficient without parallel interventions addressing glial dysfunction. More integrative approaches that modulate glial inflammation, redox capacity, and receptor signaling hold greater promise for producing durable benefits across movement disorders, psychiatric conditions, and neurodegenerative disease.

Taken together, glial-DA crosstalk has matured from speculative hypothesis into a paradigm-shifting axis in neuroscience, reframing DA as a signal conditioned by astrocytic and microglial dynamics. The next frontier lies in harnessing this framework to devise glia-targeted or glia-informed interventions that restore the cellular milieu in which DA operates, thereby promoting resilience, slowing degeneration, and advancing treatment of motivational and cognitive dysfunction across psychiatry and neurology.

## Use of AI tools declaration

The authors declare they have not used Artificial Intelligence (AI) tools in the creation of this article.

## References

[1] Luo SX, Huang EJ (2016). Dopaminergic Neurons and Brain Reward Pathways: From Neurogenesis to Circuit Assembly. Am J Pathol.

[2] Morikawa H, Paladini CA (2011). Dynamic regulation of midbrain dopamine neuron activity: intrinsic, synaptic, and plasticity mechanisms. Neuroscience.

[3] Haber SN, Behrens TE (2014). The neural network underlying incentive-based learning: implications for interpreting circuit disruptions in psychiatric disorders. Neuron.

[4] Grace AA (2016). Dysregulation of the dopamine system in the pathophysiology of schizophrenia and depression. Nat Rev Neurosci.

[5] Booth HDE, Hirst WD, Wade-Martins R (2017). The Role of Astrocyte Dysfunction in Parkinson's Disease Pathogenesis. Trends Neurosci.

[6] Li J, Serafin EK, Koorndyk N (2024). Astrocyte D1/D5 Dopamine Receptors Govern Non-Hebbian Long-Term Potentiation at Sensory Synapses onto Lamina I Spinoparabrachial Neurons. J Neurosci.

[7] Guttenplan KA, Maxwell I, Santos E (2025). GPCR signaling gates astrocyte responsiveness to neurotransmitters and control of neuronal activity. Science.

[8] Santos DE, Silva Lima SA, Moreira LS (2025). New perspectives on heterogeneity in astrocyte reactivity in neuroinflammation. Brain Behav Immun Health.

[9] Favetta G, Bubacco L (2025). Beyond neurons: How does dopamine signaling impact astrocytic functions and pathophysiology?. Prog Neurobiol.

[10] Stedehouder J, Roberts BM, Raina S (2024). Rapid modulation of striatal cholinergic interneurons and dopamine release by satellite astrocytes. Nat Commun.

[11] Frost JL, Schafer DP (2016). Microglia: Architects of the Developing Nervous System. Trends Cell Biol.

[12] She K, Yuan N, Huang M (2026). Emerging role of microglia in the developing dopaminergic system: Perturbation by early life stress. Neural Regen Res.

[13] Matt SM, Nolan R, Manikandan S (2025). Dopamine–driven increase in IL-1beta in myeloid cells is mediated by differential dopamine receptor expression and exacerbated by HIV. J Neuroinflammation.

[14] Gullotta GS, Costantino G, Sortino MA (2023). Microglia and the Blood-Brain Barrier: An External Player in Acute and Chronic Neuroinflammatory Conditions. Int J Mol Sci.

[15] Hasel P, Aisenberg WH, Bennett FC (2023). Molecular and metabolic heterogeneity of astrocytes and microglia. Cell Metab.

[16] Franco R, Reyes-Resina I, Navarro G (2021). Dopamine in Health and Disease: Much More Than a Neurotransmitter. Biomedicines.

[17] Belujon P, Grace AA (2015). Regulation of dopamine system responsivity and its adaptive and pathological response to stress. Proc Biol Sci.

[18] Choi H, Lee EJ, Shin JS (2023). Spatiotemporal characterization of glial cell activation in an Alzheimer's disease model by spatially resolved transcriptomics. Exp Mol Med.

[19] Marchetti B, Giachino C, Tirolo C (2022). “Reframing” dopamine signaling at the intersection of glial networks in the aged Parkinsonian brain as innate Nrf2/Wnt driver: Therapeutical implications. Aging Cell.

[20] Martel JC, Gatti McArthur S (2020). Dopamine Receptor Subtypes, Physiology and Pharmacology: New Ligands and Concepts in Schizophrenia. Front Pharmacol.

[21] Sanz-Galvez R, Falardeau D, Kolta A (2024). The role of astrocytes from synaptic to non-synaptic plasticity. Front Cell Neurosci.

[22] Yu X, Nagai J, Marti-Solano M (2020). Context-Specific Striatal Astrocyte Molecular Responses Are Phenotypically Exploitable. Neuron.

[23] Oda S, Funato H (2023). D1- and D2-type dopamine receptors are immunolocalized in pial and layer I astrocytes in the rat cerebral cortex. Front Neuroanat.

[24] Amato S, Averna M, Guidolin D (2023). Heteromerization of Dopamine D2 and Oxytocin Receptor in Adult Striatal Astrocytes. Int J Mol Sci.

[25] Ma Z, Stork T, Bergles DE (2016). Neuromodulators signal through astrocytes to alter neural circuit activity and behaviour. Nature.

[26] Guidolin D, Tortorella C, Marcoli M (2023). Modulation of Neuron and Astrocyte Dopamine Receptors via Receptor-Receptor Interactions. Pharmaceuticals (Basel).

[27] Pelassa S, Guidolin D, Venturini A (2019). A2A-D2 Heteromers on Striatal Astrocytes: Biochemical and Biophysical Evidence. Int J Mol Sci.

[28] Bezerra TO, Roque AC (2024). Dopamine facilitates the response to glutamatergic inputs in astrocyte cell models. PLoS Comput Biol.

[29] Khakh BS (2025). On astrocyte-neuron interactions: Broad insights from the striatum. Neuron.

[30] Malik AR, Willnow TE (2019). Excitatory Amino Acid Transporters in Physiology and Disorders of the Central Nervous System. Int J Mol Sci.

[31] Lazaridis IAG, Hirokane K, Choi W (2024). Striatal Astrocytes Influence Dopamine Dynamics and Behavioral State Transitions. biorxiv.

[32] Yin Y, Hu J, Wu H (2025). Astrocytic dopamine D1 receptor modulates glutamatergic transmission and synaptic plasticity in the prefrontal cortex through d-serine. Acta Pharm Sin B.

[33] Li J, Price TJ, Baccei ML (2022). D1/D5 Dopamine Receptors and mGluR5 Jointly Enable Non-Hebbian Long-Term Potentiation at Sensory Synapses onto Lamina I Spinoparabrachial Neurons. J Neurosci.

[34] Pittolo S, Yokoyama S, Willoughby DD (2022). Dopamine activates astrocytes in prefrontal cortex via alpha1-adrenergic receptors. Cell Rep.

[35] Amato S, Averna M, Farsetti E (2024). Control of Dopamine Signal in High-Order Receptor Complex on Striatal Astrocytes. Int J Mol Sci.

[36] Purushotham SS, Buskila Y (2023). Astrocytic modulation of neuronal signalling. Front Netw Physiol.

[37] Roberts BM, Lambert E, Livesey JA (2022). Dopamine Release in Nucleus Accumbens Is under Tonic Inhibition by Adenosine A(1) Receptors Regulated by Astrocytic ENT1 and Dysregulated by Ethanol. J Neurosci.

[38] Roberts BM, Doig NM, Brimblecombe KR (2020). GABA uptake transporters support dopamine release in dorsal striatum with maladaptive downregulation in a parkinsonism model. Nat Commun.

[39] Lassus B, Naude J, Faure P (2018). Glutamatergic and dopaminergic modulation of cortico-striatal circuits probed by dynamic calcium imaging of networks reconstructed in microfluidic chips. Sci Rep.

[40] Jennings A, Tyurikova O, Bard L (2017). Dopamine elevates and lowers astroglial Ca(2+) through distinct pathways depending on local synaptic circuitry. Glia.

[41] Fischer T, Scheffler P, Lohr C (2020). Dopamine-induced calcium signaling in olfactory bulb astrocytes. Sci Rep.

[42] Petroccione MA, D'Brant LY, Affinnih N (2023). Neuronal glutamate transporters control reciprocal inhibition and gain modulation in D1 medium spiny neurons. Elife.

[43] Martinez D, Rogers RC, Hermann GE (2020). Astrocytic glutamate transporters reduce the neuronal and physiological influence of metabotropic glutamate receptors in nucleus tractus solitarii. Am J Physiol Regul Integr Comp Physiol.

[44] Holly EN, Galanaugh J, Fuccillo MV (2024). Local regulation of striatal dopamine: A diversity of circuit mechanisms for a diversity of behavioral functions?. Curr Opin Neurobiol.

[45] Cervetto C, Maura G, Guidolin D (2023). Striatal astrocytic A2A-D2 receptor-receptor interactions and their role in neuropsychiatric disorders. Neuropharmacology.

[46] Requie LM, Gomez-Gonzalo M, Speggiorin M (2022). Astrocytes mediate long-lasting synaptic regulation of ventral tegmental area dopamine neurons. Nat Neurosci.

[47] Dury LC, Yde Ohki CM, Lesch KP (2025). The role of astrocytes in attention-deficit hyperactivity disorder: An update. Psychiatry Res.

[48] Inagaki R, Kita S, Niwa N (2025). Aberrant extracellular dopamine clearance in the prefrontal cortex exhibits ADHD-like behavior in NCX3 heterozygous mice. FEBS J.

[49] Zhuo Y, Luo B, Yi X (2024). Improved green and red GRAB sensors for monitoring dopaminergic activity in vivo. Nat Methods.

[50] MacDonald HJ, Kleppe R, Szigetvari PD (2024). The dopamine hypothesis for ADHD: An evaluation of evidence accumulated from human studies and animal models. Front Psychiatry.

[51] Sun F, Zhou J, Dai B (2020). Next-generation GRAB sensors for monitoring dopaminergic activity in vivo. Nat Methods.

[52] Evans WR, Baskar SS, Costa A (2024). Functional activation of dorsal striatum astrocytes improves movement deficits in hemi-parkinsonian mice. bioRxiv.

[53] Karimi MA, Ghajari A, Khademi R (2024). Efficacy of preladenant in improving motor symptoms in Parkinson's disease: A systematic review and meta-analysis. IBRO Neurosci Rep.

[54] Yu Y, Payne C, Marina N (2022). Remote and Selective Control of Astrocytes by Magnetomechanical Stimulation. Adv Sci (Weinh).

[55] Corkrum M, Araque A (2021). Astrocyte-neuron signaling in the mesolimbic dopamine system: the hidden stars of dopamine signaling. Neuropsychopharmacology.

[56] Salinas AG, Lee JO, Augustin SM (2023). Distinct sub-second dopamine signaling in dorsolateral striatum measured by a genetically-encoded fluorescent sensor. Nat Commun.

[57] Poewe W, Seppi K, Tanner CM (2017). Parkinson disease. Nat Rev Dis Primers.

[58] Kalia LV, Lang AE (2015). Parkinson's disease. Lancet.

[59] Hou L, Bao X, Zang C (2018). Integrin CD11b mediates alpha-synuclein-induced activation of NADPH oxidase through a Rho-dependent pathway. Redox Biol.

[60] Spillantini MG, Goedert M (2018). Neurodegeneration and the ordered assembly of alpha-synuclein. Cell Tissue Res.

[61] Qin J, Ma Z, Chen X (2023). Microglia activation in central nervous system disorders: A review of recent mechanistic investigations and development efforts. Front Neurol.

[62] Masuda T, Sankowski R, Staszewski O (2019). Spatial and temporal heterogeneity of mouse and human microglia at single-cell resolution. Nature.

[63] Ransohoff RM (2016). A polarizing question: do M1 and M2 microglia exist?. Nat Neurosci.

[64] Rangaraju S, Dammer EB, Raza SA (2018). Quantitative proteomics of acutely-isolated mouse microglia identifies novel immune Alzheimer's disease-related proteins. Mol Neurodegener.

[65] Lloyd AF, Martinez-Muriana A, Davis E (2024). Deep proteomic analysis of microglia reveals fundamental biological differences between model systems. Cell Rep.

[66] Jayanti S, Dalla Verde C, Tiribelli C (2023). Inflammation, Dopaminergic Brain and Bilirubin. Int J Mol Sci.

[67] Wong TS, Li G, Li S (2023). G protein-coupled receptors in neurodegenerative diseases and psychiatric disorders. Signal Transduct Target Ther.

[68] Han Q, Li W, Chen P (2024). Microglial NLRP3 inflammasome-mediated neuroinflammation and therapeutic strategies in depression. Neural Regen Res.

[69] Hanslik KL, Ulland TK (2020). The Role of Microglia and the Nlrp3 Inflammasome in Alzheimer's Disease. Front Neurol.

[70] Wang T, Nowrangi D, Yu L (2018). Activation of dopamine D1 receptor decreased NLRP3-mediated inflammation in intracerebral hemorrhage mice. J Neuroinflammation.

[71] Pike AF, Longhena F, Faustini G (2022). Dopamine signaling modulates microglial NLRP3 inflammasome activation: implications for Parkinson's disease. J Neuroinflammation.

[72] de Pablos RM, Herrera AJ, Espinosa-Oliva AM (2014). Chronic stress enhances microglia activation and exacerbates death of nigral dopaminergic neurons under conditions of inflammation. J Neuroinflammation.

[73] Wendimu MY, Hooks SB (2022). Microglia Phenotypes in Aging and Neurodegenerative Diseases. Cells.

[74] Lind-Holm Mogensen F, Seibler P, Grunewald A (2025). Microglial dynamics and neuroinflammation in prodromal and early Parkinson's disease. J Neuroinflammation.

[75] Theis H, Pavese N, Rektorova I (2024). Imaging Biomarkers in Prodromal and Earliest Phases of Parkinson's Disease. J Parkinsons Dis.

[76] Lee SH, Bae EJ, Park SJ (2025). Microglia-driven inflammation induces progressive tauopathies and synucleinopathies. Exp Mol Med.

[77] Deyell JS, Sriparna M, Ying M (2023). The Interplay between alpha-Synuclein and Microglia in alpha-Synucleinopathies. Int J Mol Sci.

[78] Perry VH, Holmes C (2014). Microglial priming in neurodegenerative disease. Nat Rev Neurol.

[79] Tansey MG, Goldberg MS (2010). Neuroinflammation in Parkinson's disease: its role in neuronal death and implications for therapeutic intervention. Neurobiol Dis.

[80] Badanjak K, Fixemer S, Smajic S (2021). The Contribution of Microglia to Neuroinflammation in Parkinson's Disease. Int J Mol Sci.

[81] Miao Y, Meng H (2024). The involvement of alpha-synucleinopathy in the disruption of microglial homeostasis contributes to the pathogenesis of Parkinson's disease. Cell Commun Signal.

[82] Li Y, Xia Y, Yin S (2021). Targeting Microglial alpha-Synuclein/TLRs/NF-kappaB/NLRP3 Inflammasome Axis in Parkinson's Disease. Front Immunol.

[83] Gerhard A (2016). TSPO imaging in parkinsonian disorders. Clin Transl Imaging.

[84] Lavisse S, Goutal S, Wimberley C (2021). Increased microglial activation in patients with Parkinson disease using [(18)F]-DPA714 TSPO PET imaging. Parkinsonism Relat Disord.

[85] Brooks NAH, Riar I, Klegeris A (2026). Mitochondrial damage-associated molecular patterns: Neuroimmunomodulators in central nervous system pathophysiology. Neural Regen Res.

[86] Deus CM, Tavares H, Beatriz M (2022). Mitochondrial Damage-Associated Molecular Patterns Content in Extracellular Vesicles Promotes Early Inflammation in Neurodegenerative Disorders. Cells.

[87] Zhu XX, Wang PJ, Chao S (2025). Transcriptomic profiling identifies ferroptosis and NF-kappaB signaling involved in alpha-dimorphecolic acid regulation of microglial inflammation. J Transl Med.

[88] Guan L, Lin L, Ma C (2025). Decoding crosstalk between neurotransmitters and alpha-synuclein in Parkinson's disease: pathogenesis and therapeutic implications. Ther Adv Neurol Disord.

[89] Kang S, Noh Y, Oh SJ (2023). Neuroprotective Effects of Aldehyde-Reducing Composition in an LPS-Induced Neuroinflammation Model of Parkinson's Disease. Molecules.

[90] Bailey HM, Cookson MR (2024). How Parkinson's Disease-Linked LRRK2 Mutations Affect Different CNS Cell Types. J Parkinsons Dis.

[91] Yoshioka Y, Sugino Y, Yamamuro A (2022). Dopamine inhibits the expression of proinflammatory cytokines of microglial cells through the formation of dopamine quinone in the mouse striatum. J Pharmacol Sci.

[92] Stokholm MG, Iranzo A, Ostergaard K (2017). Assessment of neuroinflammation in patients with idiopathic rapid-eye-movement sleep behaviour disorder: a case-control study. Lancet Neurol.

[93] Burke WJ, Li SW, Williams EA (2003). 3,4-Dihydroxyphenylacetaldehyde is the toxic dopamine metabolite in vivo: implications for Parkinson's disease pathogenesis. Brain Res.

[94] Schafer DP, Lehrman EK, Stevens B (2013). The “quad-partite” synapse: microglia-synapse interactions in the developing and mature CNS. Glia.

[95] Huo A, Wang J, Li Q (2024). Molecular mechanisms underlying microglial sensing and phagocytosis in synaptic pruning. Neural Regen Res.

[96] Soteros BM, Sia GM (2022). Complement and microglia dependent synapse elimination in brain development. WIREs Mech Dis.

[97] Loh JS, Mak WQ, Tan LKS (2024). Microbiota-gut-brain axis and its therapeutic applications in neurodegenerative diseases. Signal Transduct Target Ther.

[98] Juarez Olguin H, Calderon Guzman D, Hernandez Garcia E (2016). The Role of Dopamine and Its Dysfunction as a Consequence of Oxidative Stress. Oxid Med Cell Longev.

[99] Cai Y, Liu J, Wang B (2022). Microglia in the Neuroinflammatory Pathogenesis of Alzheimer's Disease and Related Therapeutic Targets. Front Immunol.

[100] Gao C, Jiang J, Tan Y (2023). Microglia in neurodegenerative diseases: mechanism and potential therapeutic targets. Signal Transduct Target Ther.

[101] Vainchtein ID, Chin G, Cho FS (2018). Astrocyte-derived interleukin-33 promotes microglial synapse engulfment and neural circuit development. Science.

[102] Sun M, You H, Hu X (2023). Microglia-Astrocyte Interaction in Neural Development and Neural Pathogenesis. Cells.

[103] Spreng AS, Brull M, Leisner H (2022). Distinct and Dynamic Transcriptome Adaptations of iPSC-Generated Astrocytes after Cytokine Stimulation. Cells.

[104] Jiwaji Z, Tiwari SS, Aviles-Reyes RX (2022). Reactive astrocytes acquire neuroprotective as well as deleterious signatures in response to Tau and Ass pathology. Nat Commun.

[105] Naffaa MM (2025). Mechanisms of astrocytic and microglial purinergic signaling in homeostatic regulation and implications for neurological disease. Explor Neurosci.

[106] Lindberg D, Shan D, Ayers-Ringler J (2015). Purinergic signaling and energy homeostasis in psychiatric disorders. Curr Mol Med.

[107] Carracedo S, Launay A, Dechelle-Marquet PA (2024). Purinergic-associated immune responses in neurodegenerative diseases. Prog Neurobiol.

[108] Pawelec P, Ziemka-Nalecz M, Sypecka J (2020). The Impact of the CX3CL1/CX3CR1 Axis in Neurological Disorders. Cells.

[109] Chu Y, Harms AS, Boehringer A (2025). Decreased neuronal and increased endothelial fractalkine expression are associated with neuroinflammation in Parkinson's disease and related disorders. Front Cell Neurosci.

[110] Prada I, Gabrielli M, Turola E (2018). Glia-to-neuron transfer of miRNAs via extracellular vesicles: a new mechanism underlying inflammation-induced synaptic alterations. Acta Neuropathol.

[111] Pistono C, Bister N, Stanova I (2020). Glia-Derived Extracellular Vesicles: Role in Central Nervous System Communication in Health and Disease. Front Cell Dev Biol.

[112] Marchetti B, Leggio L, L'Episcopo F (2020). Glia-Derived Extracellular Vesicles in Parkinson's Disease. J Clin Med.

[113] Nagai J, Yu X, Papouin T (2021). Behaviorally consequential astrocytic regulation of neural circuits. Neuron.

[114] Agarwal D, Sandor C, Volpato V (2020). A single-cell atlas of the human substantia nigra reveals cell-specific pathways associated with neurological disorders. Nat Commun.

[115] Fornari Laurindo L, Aparecido Dias J, Cressoni Araujo A (2023). Immunological dimensions of neuroinflammation and microglial activation: exploring innovative immunomodulatory approaches to mitigate neuroinflammatory progression. Front Immunol.

[116] Ma M, Paryani F, Jakubiak K (2025). The spatial landscape of glial pathology and T cell response in Parkinson's disease substantia nigra. Nat Commun.

[117] Gaertner Z, Oram C, Schneeweis A (2025). Molecular and spatial transcriptomic classification of midbrain dopamine neurons and their alterations in a LRRK2(G2019S) model of Parkinson's disease. Elife.

[118] Rademacher K, Doric Z, Haddad D (2025). Chronic hyperactivation of midbrain dopamine neurons causes preferential dopamine neuron degeneration. Elife.

[119] Gao MY, Wang JQ, He J (2023). Single-Cell RNA-Sequencing in Astrocyte Development, Heterogeneity, and Disease. Cell Mol Neurobiol.

[120] Khan ZU, Koulen P, Rubinstein M (2001). An astroglia-linked dopamine D2-receptor action in prefrontal cortex. Proc Natl Acad Sci U S A.

[121] Rahimian R, Belliveau C, Chen R (2022). Microglial Inflammatory-Metabolic Pathways and Their Potential Therapeutic Implication in Major Depressive Disorder. Front Psychiatry.

[122] Kopec AM, Smith CJ, Ayre NR (2018). Microglial dopamine receptor elimination defines sex-specific nucleus accumbens development and social behavior in adolescent rats. Nat Commun.

[123] Furuyashiki T, Kitaoka S (2019). Neural mechanisms underlying adaptive and maladaptive consequences of stress: Roles of dopaminergic and inflammatory responses. Psychiatry Clin Neurosci.

[124] Jimenez-Gonzalez A, Gomez-Acevedo C, Ochoa-Aguilar A (2022). The Role of Glia in Addiction: Dopamine as a Modulator of Glial Responses in Addiction. Cell Mol Neurobiol.

[125] Lee HG, Wheeler MA, Quintana FJ (2022). Function and therapeutic value of astrocytes in neurological diseases. Nat Rev Drug Discov.

[126] Mastroeni D, Grover A, Leonard B (2009). Microglial responses to dopamine in a cell culture model of Parkinson's disease. Neurobiol Aging.

[127] Mao S, Qiao R, Wang Q (2025). Activity and Heterogeneity of Astrocytes in Neurological Diseases: Molecular Mechanisms and Therapeutic Targets. MedComm (2020).

[128] Yoo S LK, Seo J, Choi H (2024). Single-nucleus and spatial transcriptomic analysis identified molecular features of neuronal heterogeneity and distinct glial responses in Parkinson's disease. biorxiv.

[129] Hu N, Chen L, Hu G (2025). Advancements in extracellular vesicle therapy for neurodegenerative diseases. Explor Neuroprotective Ther.

[130] Felger JC, Li Z, Haroon E (2016). Inflammation is associated with decreased functional connectivity within corticostriatal reward circuitry in depression. Mol Psychiatry.

[131] Felger JC, Miller AH (2012). Cytokine effects on the basal ganglia and dopamine function: the subcortical source of inflammatory malaise. Front Neuroendocrinol.

[132] Sugama S, Takenouchi T, Hashimoto M (2019). Stress-induced microglial activation occurs through beta-adrenergic receptor: noradrenaline as a key neurotransmitter in microglial activation. J Neuroinflammation.

[133] Tujula I, Hyvarinen T, Lotila J (2025). Modeling neuroinflammatory interactions between microglia and astrocytes in a human iPSC-based coculture platform. Cell Commun Signal.

[134] Iring A TA, Baranyi M, Otrokocsi L (2021). Central inhibition of P2Y12R differentially regulates survival and neuronal loss in MPTP-induced Parkinsonism in mice. biorxiv.

[135] Murphy-Royal C, Ching S, Papouin T (2023). A conceptual framework for astrocyte function. Nat Neurosci.

[136] Stowell R, Wang KH (2025). Dopaminergic signaling regulates microglial surveillance and adolescent plasticity in the mouse frontal cortex. Nat Commun.

[137] Alcaro A, Huber R, Panksepp J (2007). Behavioral functions of the mesolimbic dopaminergic system: an affective neuroethological perspective. Brain Res Rev.

[138] Baik JH (2020). Stress and the dopaminergic reward system. Exp Mol Med.

[139] Ortinski PI, Reissner KJ, Turner J (2022). Control of complex behavior by astrocytes and microglia. Neurosci Biobehav Rev.

[140] Zhou ZC, Gordon-Fennell A, Piantadosi SC (2023). Deep-brain optical recording of neural dynamics during behavior. Neuron.

[141] Serra I, Martin-Monteagudo C, Sanchez Romero J (2025). Astrocyte ensembles manipulated with AstroLight tune cue-motivated behavior. Nat Neurosci.

[142] Kang S, Hong SI, Kang S (2023). Astrocyte activities in the external globus pallidus regulate action-selection strategies in reward-seeking behaviors. Sci Adv.

[143] Yang MA, Kang S, Hong SI (2025). Astrocytes in the External Globus Pallidus Selectively Represent Routine Formation During Repeated Reward-Seeking in Mice. eNeuro.

[144] Zhang C, Dulinskas R, Ineichen C (2024). Chronic stress deficits in reward behaviour co-occur with low nucleus accumbens dopamine activity during reward anticipation specifically. Commun Biol.

[145] Park K, Lee SJ (2020). Deciphering the star codings: astrocyte manipulation alters mouse behavior. Exp Mol Med.

[146] Nowak DB, Taborda-Bejarano JP, Chaure FJ (2025). Understanding Microglia in Mesocorticolimbic Circuits: Implications for the Study of Chronic Stress and Substance Use Disorders. Cells.

[147] da Silva MCM, Iglesias LP, Candelario-Jalil E (2023). Role of Microglia in Psychostimulant Addiction. Curr Neuropharmacol.

[148] Avolio E, Fazzari G, Mele M (2017). Unpredictable Chronic Mild Stress Paradigm Established Effects of Pro- and Anti-inflammatory Cytokine on Neurodegeneration-Linked Depressive States in Hamsters with Brain Endothelial Damages. Mol Neurobiol.

[149] Farooq RK, Isingrini E, Tanti A (2012). Is unpredictable chronic mild stress (UCMS) a reliable model to study depression-induced neuroinflammation?. Behav Brain Res.

[150] Xia X, Li K, Zou W (2025). The central role of microglia in major depressive disorder and its potential as a therapeutic target. Front Behav Neurosci.

[151] Paganin W, Signorini S (2024). Inflammatory biomarkers in depression: scoping review. BJPsych Open.

[152] Reemst K, Kracht L, Kotah JM (2022). Early-life stress lastingly impacts microglial transcriptome and function under basal and immune-challenged conditions. Transl Psychiatry.

[153] Jamil S, Raza ML, Moradikor N (2025). Early life stress and brain development: Neurobiological and behavioral effects of chronic stress. Prog Brain Res.

[154] Gongwer MW, Etienne F, Moca EN (2025). Microglia regulate nucleus accumbens synaptic development and circuit function underlying threat avoidance behaviors. Res Sq.

[155] Smith JA, Das A, Ray SK (2012). Role of pro-inflammatory cytokines released from microglia in neurodegenerative diseases. Brain Res Bull.

[156] Lull ME, Block ML (2010). Microglial activation and chronic neurodegeneration. Neurotherapeutics.

[157] Thrailkill EA, Daniels CW (2024). The temporal structure of goal-directed and habitual operant behavior. J Exp Anal Behav.

[158] Delgado L, Navarrete M (2023). Shining the Light on Astrocytic Ensembles. Cells.

[159] Shirokova OM, Kuzmina DM, Zaborskaya OG (2025). The Long-Term Effects of Chronic Unpredictable Mild Stress Experienced During Adolescence Could Vary Depending on Biological Sex. Int J Mol Sci.

[160] Bergamini G, Mechtersheimer J, Azzinnari D (2018). Chronic social stress induces peripheral and central immune activation, blunted mesolimbic dopamine function, and reduced reward-directed behaviour in mice. Neurobiol Stress.

[161] Nusslock R, Alloy LB, Brody GH (2024). Annual Research Review: Neuroimmune network model of depression: a developmental perspective. J Child Psychol Psychiatry.

[162] Cervetto C, Venturini A, Guidolin D (2018). Homocysteine and A2A-D2 Receptor-Receptor Interaction at Striatal Astrocyte Processes. J Mol Neurosci.

[163] Wright WJ, Dong Y (2020). Psychostimulant-Induced Adaptations in Nucleus Accumbens Glutamatergic Transmission. Cold Spring Harb Perspect Med.

[164] Williams Ph DM, Cox B, Lafuse Ph DW (2019). Epstein-Barr Virus dUTPase Induces Neuroinflammatory Mediators: Implications for Myalgic Encephalomyelitis/Chronic Fatigue Syndrome. Clin Ther.

[165] Dobryakova E, Genova HM, DeLuca J (2015). The dopamine imbalance hypothesis of fatigue in multiple sclerosis and other neurological disorders. Front Neurol.

[166] Volkow ND, Wang GJ, Newcorn JH (2011). Motivation deficit in ADHD is associated with dysfunction of the dopamine reward pathway. Mol Psychiatry.

[167] Vandenbark AA, Offner H, Matejuk S (2021). Microglia and astrocyte involvement in neurodegeneration and brain cancer. J Neuroinflammation.

[168] Kanthasamy A, Jin H, Charli A (2019). Environmental neurotoxicant-induced dopaminergic neurodegeneration: a potential link to impaired neuroinflammatory mechanisms. Pharmacol Ther.

[169] Wang J, Wang F, Mai D (2020). Molecular Mechanisms of Glutamate Toxicity in Parkinson's Disease. Front Neurosci.

[170] Kamath T, Abdulraouf A, Burris SJ (2022). Single-cell genomic profiling of human dopamine neurons identifies a population that selectively degenerates in Parkinson's disease. Nat Neurosci.

[171] Sportelli L, Eisenberg DP, Passiatore R (2024). Dopamine signaling enriched striatal gene set predicts striatal dopamine synthesis and physiological activity in vivo. Nat Commun.

[172] Voicu V, Toader C, Serban M (2025). Systemic Neurodegeneration and Brain Aging: Multi-Omics Disintegration, Proteostatic Collapse, and Network Failure Across the CNS. Biomedicines.

[173] Stevenson R, Samokhina E, Rossetti I (2020). Neuromodulation of Glial Function During Neurodegeneration. Front Cell Neurosci.

[174] Jiang Q, Liu J, Huang S (2025). Antiageing strategy for neurodegenerative diseases: from mechanisms to clinical advances. Signal Transduct Target Ther.

[175] Li K, Li J, Zheng J (2019). Reactive Astrocytes in Neurodegenerative Diseases. Aging Dis.

[176] Lee HG, Lee JH, Flausino LE (2023). Neuroinflammation: An astrocyte perspective. Sci Transl Med.

[177] Liddelow SA, Guttenplan KA, Clarke LE (2017). Neurotoxic reactive astrocytes are induced by activated microglia. Nature.

[178] Garland EF, Hartnell IJ, Boche D (2022). Microglia and Astrocyte Function and Communication: What Do We Know in Humans?. Front Neurosci.

[179] Zhang Z, Niu K, Huang T (2025). Microglia depletion reduces neurodegeneration and remodels extracellular matrix in a mouse Parkinson's disease model triggered by alpha-synuclein overexpression. NPJ Parkinsons Dis.

[180] Thi Lai T, Kim YE, Nguyen LTN (2024). Microglial inhibition alleviates alpha-synuclein propagation and neurodegeneration in Parkinson's disease mouse model. NPJ Parkinsons Dis.

[181] Sunna S, Bowen CA, Ramelow CC (2023). Advances in proteomic phenotyping of microglia in neurodegeneration. Proteomics.

[182] Li Z, Xu P, Deng Y (2024). M1 Microglia-Derived Exosomes Promote A1 Astrocyte Activation and Aggravate Ischemic Injury via circSTRN3/miR-331-5p/MAVS/NF-kappaB Pathway. J Inflamm Res.

[183] Guo M, Guan A, Zhang M (2025). Exosome-mediated microglia-astrocyte interactions drive neuroinflammation in Parkinson's disease with Peli1 as a potential therapeutic target. Pharmacol Res.

[184] Buoso C, Seifert M, Lang M (2024). Dopamine‑iron homeostasis interaction rescues mitochondrial fitness in Parkinson's disease. Neurobiol Dis.

[185] Nicholson KJ, Gilliland TM, Winkelstein BA (2014). Upregulation of GLT-1 by treatment with ceftriaxone alleviates radicular pain by reducing spinal astrocyte activation and neuronal hyperexcitability. J Neurosci Res.

[186] Abulseoud OA, Alasmari F, Hussein AM (2022). Ceftriaxone as a Novel Therapeutic Agent for Hyperglutamatergic States: Bridging the Gap Between Preclinical Results and Clinical Translation. Front Neurosci.

[187] Jiao L, Li X, Luo Y (2022). Iron metabolism mediates microglia susceptibility in ferroptosis. Front Cell Neurosci.

[188] Ru Q, Li Y, Chen L (2024). Iron homeostasis and ferroptosis in human diseases: mechanisms and therapeutic prospects. Signal Transduct Target Ther.

[189] Rudroff T (2024). Decoding Post-Viral Fatigue: The Basal Ganglia's Complex Role in Long-COVID. Neurol Int.

[190] Khan S AY, Kazi N, Sideeque S (2025). Brain structural and functional alteration in movement disorders: A narrative review of markers and their dynamic changes. NeuroMarkers.

[191] Mancini M, Natoli S, Gardoni F (2023). Dopamine Transmission Imbalance in Neuroinflammation: Perspectives on Long-Term COVID-19. Int J Mol Sci.

[192] Adamu A, Li S, Gao F (2024). The role of neuroinflammation in neurodegenerative diseases: current understanding and future therapeutic targets. Front Aging Neurosci.

[193] Kanberg N, Simren J, Eden A (2021). Neurochemical signs of astrocytic and neuronal injury in acute COVID-19 normalizes during long-term follow-up. EBioMedicine.

[194] Vrettou CS, Vassiliou AG, Keskinidou C (2024). A Prospective Study on Neural Biomarkers in Patients with Long-COVID Symptoms. J Pers Med.

[195] Picca A, Ferri E, Calvani R (2022). Age-Associated Glia Remodeling and Mitochondrial Dysfunction in Neurodegeneration: Antioxidant Supplementation as a Possible Intervention. Nutrients.

[196] Trist BG, Hare DJ, Double KL (2019). Oxidative stress in the aging substantia nigra and the etiology of Parkinson's disease. Aging Cell.

[197] Zhang S, Zhang C, Zhang Y (2025). Unraveling the role of neuregulin-mediated astrocytes-OPCs axis in the pathogenesis of age-related macular degeneration and Parkinson's disease. Sci Rep.

[198] Iba M, Lee YJ, Horan-Portelance L (2025). Microglial and neuronal fates following inhibition of CSF-1R in synucleinopathy mouse model. Brain Behav Immun.

[199] Stoll AC, Kemp CJ, Patterson JR (2024). Alpha-synuclein inclusion responsive microglia are resistant to CSF1R inhibition. J Neuroinflammation.

[200] Eo H, Kim S, Jung UJ (2024). Alpha-Synuclein and Microglia in Parkinson's Disease: From Pathogenesis to Therapeutic Prospects. J Clin Med.

[201] Guo M, Wang J, Zhao Y (2020). Microglial exosomes facilitate alpha-synuclein transmission in Parkinson's disease. Brain.

[202] Jiao D, Yang Y, Wang K (2025). Ferroptosis: a novel pathogenesis and therapeutic strategies for Parkinson disease: A review. Medicine (Baltimore).

[203] Tang S, Zhang J, Chen J (2025). Ferroptosis in neurodegenerative diseases: potential mechanisms of exercise intervention. Front Cell Dev Biol.

[204] Zhi H, Wang X, Chen Y (2025). Ceftriaxone affects ferroptosis and alleviates glial cell activation in Parkinson's disease. Int J Mol Med.

[205] Chotibut T, Meadows S, Kasanga EA (2017). Ceftriaxone reduces L-dopa-induced dyskinesia severity in 6-hydroxydopamine parkinson's disease model. Mov Disord.

[206] Greene C, Connolly R, Brennan D (2024). Blood-brain barrier disruption and sustained systemic inflammation in individuals with long COVID-associated cognitive impairment. Nat Neurosci.

[207] Popa E, Popa AE, Poroch M (2025). The Molecular Mechanisms of Cognitive Dysfunction in Long COVID: A Narrative Review. Int J Mol Sci.

[208] Bhatia TN, Jamenis AS, Abbas M (2023). A 14-day pulse of PLX5622 modifies alpha-synucleinopathy in preformed fibril-infused aged mice of both sexes. Neurobiol Dis.

[209] Yun SP, Kam TI, Panicker N (2018). Block of A1 astrocyte conversion by microglia is neuroprotective in models of Parkinson's disease. Nat Med.

[210] Li M, Chen M, Li H (2024). Glial cells improve Parkinson's disease by modulating neuronal function and regulating neuronal ferroptosis. Front Cell Dev Biol.

[211] Chen X, Zhang G, Liu M (2025). New perspectives on molecular mechanisms underlying exercise-induced benefits in Parkinson's disease. NPJ Parkinsons Dis.

[212] Luthra NS, Mehta N, Munoz MJ (2025). Aerobic exercise-induced changes in fluid biomarkers in Parkinson's disease. NPJ Parkinsons Dis.

[213] Hein ZM, Thazin, Kumar S (2025). Immunomodulatory Mechanisms Underlying Neurological Manifestations in Long COVID: Implications for Immune-Mediated Neurodegeneration. Int J Mol Sci.

[214] Huang M, Long A, Hao L (2025). Astrocyte in Neurological Disease: Pathogenesis and Therapy. MedComm (2020).

[215] Todd AC, Hardingham GE (2020). The Regulation of Astrocytic Glutamate Transporters in Health and Neurodegenerative Diseases. Int J Mol Sci.

[216] Naffaa MM (2025). Monoamine Oxidase B in Astrocytic GABA Synthesis: A Central Mechanism in Neurodegeneration and Neuroinflammation. J Cell Signal.

[217] Villemagne VL, Harada R, Dore V (2022). First-in-Humans Evaluation of (18)F-SMBT-1, a Novel (18)F-Labeled Monoamine Oxidase-B PET Tracer for Imaging Reactive Astrogliosis. J Nucl Med.

[218] Bellaver B, Povala G, Ferreira PCL (2023). Astrocyte reactivity influences amyloid-beta effects on tau pathology in preclinical Alzheimer's disease. Nat Med.

[219] Kong Y, Cao L, Wang J (2024). In vivo reactive astrocyte imaging using [(18)F]SMBT-1 in tauopathy and familial Alzheimer's disease mouse models: A multi-tracer study. J Neurol Sci.

[220] Chatzipieris FP, Kokkalis A, Georgiou N (2025). New Prospects in the Inhibition of Monoamine Oxidase‑B (MAO-B) Utilizing Propargylamine Derivatives for the Treatment of Alzheimer's Disease: A Review. ACS Omega.

[221] Zou DJ, Liu RZ, Lv YJ (2024). Chromone-deferiprone hybrids as novel MAO-B inhibitors and iron chelators for the treatment of Alzheimer's disease. Org Biomol Chem.

[222] Duta C, Muscurel C, Dogaru CB (2024). Ferroptosis-A Shared Mechanism for Parkinson's Disease and Type 2 Diabetes. Int J Mol Sci.

[223] Liu S, Gao X, Zhou S (2022). New Target for Prevention and Treatment of Neuroinflammation: Microglia Iron Accumulation and Ferroptosis. ASN Neuro.

[224] Xu Y, Jia B, Li J (2024). The Interplay between Ferroptosis and Neuroinflammation in Central Neurological Disorders. Antioxidants (Basel).

[225] Feng S, Tang D, Wang Y (2023). The mechanism of ferroptosis and its related diseases. Mol Biomed.

[226] Oh SJ, Ahn H, Jung KH (2020). Evaluation of the Neuroprotective Effect of Microglial Depletion by CSF-1R Inhibition in a Parkinson's Animal Model. Mol Imaging Biol.

[227] Ho MS (2025). Clearance Pathways for alpha-Synuclein in Parkinson's Disease. J Neurochem.

[228] Basilico B, Ferrucci L, Khan A (2022). What microglia depletion approaches tell us about the role of microglia on synaptic function and behavior. Front Cell Neurosci.

[229] Adaikkan C RIM, Lorenzo Bozzelli P, Sears M (2025). A multimodal approach of microglial CSF1R inhibition and GENUS provides therapeutic effects in Alzheimer's disease mice. bioRxiv.

[230] Noh MY, Kwon HS, Kwon MS (2025). Biomarkers and therapeutic strategies targeting microglia in neurodegenerative diseases: current status and future directions. Mol Neurodegener.

[231] Taguchi D, Ehara A, Kadowaki T (2020). Minocycline Alleviates Cluster Formation of Activated Microglia and Age-dependent Dopaminergic Cell Death in the Substantia Nigra of Zitter Mutant Rat. Acta Histochem Cytochem.

[232] Griffin JM, Fackelmeier B, Fong DM (2019). Astrocyte-selective AAV gene therapy through the endogenous GFAP promoter results in robust transduction in the rat spinal cord following injury. Gene Ther.

[233] Van Den Herrewegen Y, Sanderson TM, Sahu S (2021). Side-by-side comparison of the effects of Gq- and Gi-DREADD-mediated astrocyte modulation on intracellular calcium dynamics and synaptic plasticity in the hippocampal CA1. Mol Brain.

[234] Zhong X, Gu H, Lim J (2025). Genetically encoded sensors illuminate in vivo detection for neurotransmission: Development, application, and optimization strategies. IBRO Neurosci Rep.

[235] Harada R, Hayakawa Y, Ezura M (2021). (18)F-SMBT-1: A Selective and Reversible PET Tracer for Monoamine Oxidase-B Imaging. J Nucl Med.

[236] Mishra S, Gordon BA, Su Y (2017). AV-1451 PET imaging of tau pathology in preclinical Alzheimer disease: Defining a summary measure. Neuroimage.

[237] Zhang M, Qian XH, Hu J (2024). Integrating TSPO PET imaging and transcriptomics to unveil the role of neuroinflammation and amyloid-beta deposition in Alzheimer's disease. Eur J Nucl Med Mol Imaging.

[238] Bonomi CG, Chiaravalloti A, Camedda R (2023). Functional Correlates of Microglial and Astrocytic Activity in Symptomatic Sporadic Alzheimer's Disease: A CSF/(18)F-FDG-PET Study. Biomedicines.

[239] Ballweg A, Klaus C, Vogler L (2023). [(18)F]F-DED PET imaging of reactive astrogliosis in neurodegenerative diseases: preclinical proof of concept and first-in-human data. J Neuroinflammation.

[240] Lin J, Ou R, Li C (2023). Plasma glial fibrillary acidic protein as a biomarker of disease progression in Parkinson's disease: a prospective cohort study. BMC Med.

[241] Shi Q, Gutierrez RA, Bhat MA (2025). Microglia, Trem2, and Neurodegeneration. Neuroscientist.

[242] Zhang L, Xiang X, Li Y (2025). TREM2 and sTREM2 in Alzheimer's disease: from mechanisms to therapies. Mol Neurodegener.

[243] Gabrielli M, Raffaele S, Fumagalli M (2022). The multiple faces of extracellular vesicles released by microglia: Where are we 10 years after?. Front Cell Neurosci.

[244] Wang P, Lan G, Xu B (2023). alpha-Synuclein-carrying astrocytic extracellular vesicles in Parkinson pathogenesis and diagnosis. Transl Neurodegener.

[245] Kopp KO, Glotfelty EJ, Li Y (2022). Glucagon-like peptide-1 (GLP-1) receptor agonists and neuroinflammation: Implications for neurodegenerative disease treatment. Pharmacol Res.

[246] Cheng D, Yang S, Zhao X (2022). The Role of Glucagon-Like Peptide-1 Receptor Agonists (GLP-1 RA) in Diabetes-Related Neurodegenerative Diseases. Drug Des Devel Ther.

[247] Timper K, Del Rio-Martin A, Cremer AL (2020). GLP-1 Receptor Signaling in Astrocytes Regulates Fatty Acid Oxidation, Mitochondrial Integrity, and Function. Cell Metab.

[248] Bayram E, Batzu L, Tilley B (2023). Clinical trials for cognition in Parkinson's disease: Where are we and how can we do better?. Parkinsonism Relat Disord.

[249] Diz-Chaves Y, Mastoor Z, Spuch C (2022). Anti-Inflammatory Effects of GLP-1 Receptor Activation in the Brain in Neurodegenerative Diseases. Int J Mol Sci.

[250] Fan R, Xu F, Previti ML (2007). Minocycline reduces microglial activation and improves behavioral deficits in a transgenic model of cerebral microvascular amyloid. J Neurosci.

[251] Sriram K, Miller DB, O'Callaghan JP (2006). Minocycline attenuates microglial activation but fails to mitigate striatal dopaminergic neurotoxicity: role of tumor necrosis factor-alpha. J Neurochem.

